# Control of translation during the unfolded protein response in maize seedlings: Life without PERKs

**DOI:** 10.1002/pld3.241

**Published:** 2020-07-30

**Authors:** Pulkit Kanodia, Paramasivan Vijayapalani, Renu Srivastava, Ran Bi, Peng Liu, W. Allen Miller, Stephen H. Howell

**Affiliations:** ^1^ Plant Pathology and Microbiology Department Iowa State University Ames IA USA; ^2^ Interdepartmental Genetics and Genomics Major Iowa State University Ames IA USA; ^3^ Plant Science Institute Iowa State University Ames IA USA; ^4^ Statistics Department Iowa State University Ames IA USA; ^5^ Genetics, Development and Cell Biology Department Iowa State University Ames IA USA

**Keywords:** ER stress, protein synthesis, ribosome profiling, ribosome‐protected fragments, stress granules, translation efficiency

## Abstract

The accumulation of misfolded proteins in the endoplasmic reticulum (ER) defines a condition called ER stress that induces the unfolded protein response (UPR). The UPR in mammalian cells attenuates protein synthesis initiation, which prevents the piling up of misfolded proteins in the ER. Mammalian cells rely on Protein Kinase RNA‐Like Endoplasmic Reticulum Kinase (PERK) phosphorylation of eIF2α to arrest protein synthesis, however, plants do not have a PERK homolog, so the question is whether plants control translation in response to ER stress. We compared changes in RNA levels in the transcriptome to the RNA levels protected by ribosomes and found a decline in translation efficiency, including many UPR genes, in response to ER stress. The decline in translation efficiency is due to the fact that many mRNAs are not loaded onto polyribosomes (polysomes) in proportion to their increase in total RNA, instead some of the transcripts accumulate in stress granules (SGs). The RNAs that populate SGs are not derived from the disassembly of polysomes because protein synthesis remains steady during stress. Thus, the surge in transcription of UPR genes in response to ER stress is accompanied by the formation of SGs, and the sequestration of mRNAs in SGs may serve to temporarily relieve the translation load during ER stress.

## 1 INTRODUCTION

A major challenge for plant scientists is to understand how plants adapt to climate change given the prediction for greater weather extremes in the future. Adverse environmental conditions, such as elevated temperatures, tend to exacerbate error‐prone processes in plants, such as the folding of proteins in the endoplasmic reticulum (ER) (Howell, 2013; Nakajima & Suzuki, 2013; Strasser, 2018). Errors in protein folding lead to the accumulation of misfolded proteins, a potentially toxic condition termed “ER stress” (Walter & Ron, 2011). ER stress induces an adaptive response called the unfolded protein response (UPR) which helps to mitigate the damage caused by stress and to protect plants from further stress. The UPR upregulates the expression of genes that aid in protein import, folding, quality control, and export (Iwata & Koizumi, 2005; Kamauchi, Nakatani, Nakano, & Urade, 2005; Martinez & Chrispeels, 2003; Srivastava et al., 2018).

**FIGURE 1:**
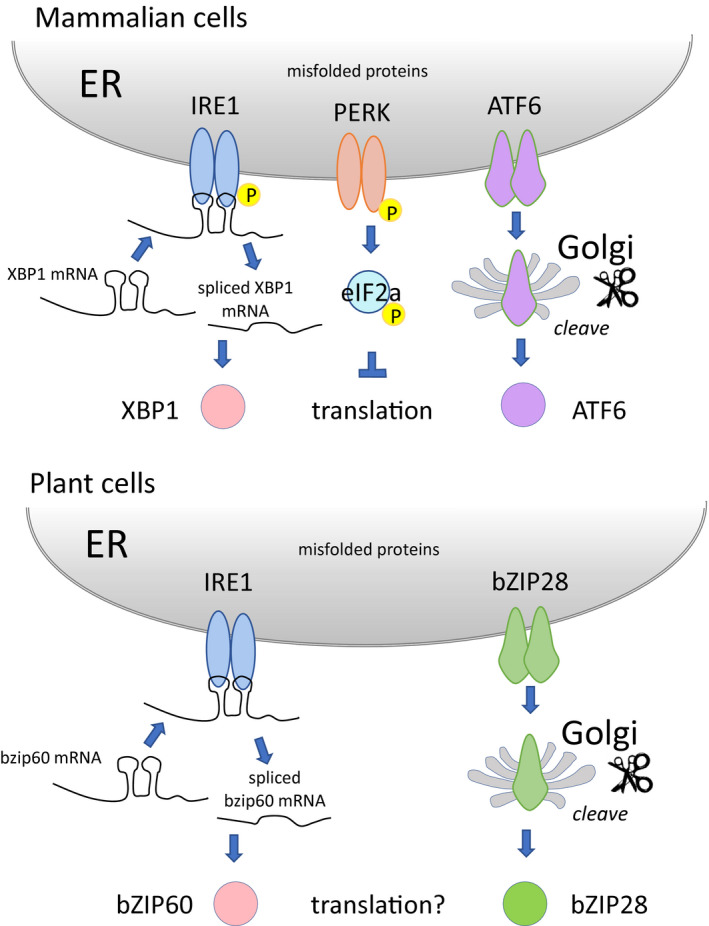
Differences between the UPR in mammalian and plant cells. (a) Mammalian cells have three arms of the UPR signaling pathway including an arm involving Protein Kinase RNA‐Like Endoplasmic Reticulum Kinase (PERK) which phosphorylates eIF2α in response to ER stress, thereby inhibiting translation initiation. (b) Plant cells have two arms of the UPR signaling pathway and do not have a PERK homolog leaving open the question as to whether translation is attenuated in response to ER stress in plants. Not shown is the RIDD activity of IRE1 in which this factor attacks other mRNAs on the ER membrane. ER, endoplasmic reticulum; RIDD, regulated IRE1‐dependent decay; UPR, unfolded protein response

The UPR is highly conserved among eukaryotic organisms, and in mammalian cells the UPR leads to an attenuation in protein synthesis initiation (Harding, Novoa, et al., 2000). The slow down in protein synthesis lightens the load for the protein folding machinery and prevents further buildup of misfolded proteins in the ER. Translation initiation in mammalian cells is attenuated by the action of Protein Kinase RNA‐Like Endoplasmic Reticulum Kinase (PERK) (Yan et al., 2002), an ER membrane enzyme which is activated by ER stress to phosphorylate the translation initiation factor eIF2α (Harding, Novoa, et al., 2000). Phosphorylation of eIF2α inhibits the guanine nucleotide exchange factor eIF2B and thus downregulates the global protein synthesis (Clemens, 2001). PERK is a key component in one of three arms of the UPR signaling pathway in mammalian cells (Yan et al., 2002). Of the other two arms, one involves membrane‐associated bZIP transcription factors, such as ATF6, which are mobilized and relocated to the nucleus in response to ER stress, while the other arm consists of the RNA splicing factor IRE1 that splices XBP1 mRNA in response to stress (Figure 1)

Plants have only two arms of the UPR signaling pathway (Howell, 2013). Similar to mammalian cells, plants, such as Arabidopsis, have an arm of the pathway comprised of membrane‐associated transcription factors bZIP17 and bZIP28, and an arm involving IRE1, which in the case of plants splices the mRNA for bZIP60 (Deng et al., 2011; Nagashima et al., 2011). Plants do not have a PERK homolog, which leads one to ask whether plants control translation in response to ER stress. This is a critical issue because PERK is essential for translational regulation and cell survival in mammalian cells (Harding, Zhang, Bertolotti, Zeng, & Ron, 2000). Arabidopsis has an eIF2α homolog that is phosphorylated in response to various stresses, including ER stress (Izquierdo et al., 2018; Zhang et al., 2008). However, the phosphorylation of eIF2α in response to ER stress in Arabidopsis is GCN2 dependent (Zhang et al., 2008). GCN2 is not an ER factor, and so it is not understood how it is activated by ER stress and whether eIF2α phosphorylation affects translation in plants (Browning & Bailey‐Serres, 2015). Arabidopsis eIF2α is also phosphorylated following treatment with herbicides, such as glyphosate or chlorsulfuron, that block amino acid biosynthesis (Lageix et al., 2008; Zhang et al., 2008).

Another means by which ER stress can attenuate general protein synthesis is through the activation of regulated IRE1‐dependent decay of mRNA (RIDD) (Hollien & Weissman, 2006). RIDD involves the promiscuous attack by IRE1’s RNase activity on RNA substrates other than its normal splicing targets. In Drosophila, IRE1 attacks a wide range of substrates consisting of mRNAs encoding secreted proteins (Gaddam, Stevens, & Hollien, 2013). However, in mammalian cells RIDD is more specific, attacking mRNAs with hairpin structures and core sequences comparable to those found in its normal RNA splicing target, XBP1 (Gaddam et al., 2013; Kadowaki & Nishitoh, 2019; Moore & Hollien, 2015). RIDD has also been observed in plants, and in Arabidopsis, RIDD has a wide range of RNA targets encoding secreted proteins (Mishiba et al., 2013). In maize, RIDD appears to have some preferred targets including the mRNAs encoding a family of secreted peroxidases (Srivastava et al., 2018). However, the extent of RIDD activity has not been assessed in maize to determine whether RIDD would have a general effect on translation in response to ER stress.

In this study, we subjected maize seedlings to ER stress and determined the effect on translation. This study was preceded by another in which we reported changes in the transcriptome in response to ER stress induced by tunicamycin (TM) treatment (Srivastava et al., 2018). Most notable among the events was a burst in canonical UPR gene expression from 6 to 12 hr following the imposition of stress. The fate of these RNA transcripts during the burst in synthesis is not known. A point of interest is whether these UPR transcripts as well as others are translated and thus present on polyribosomes (polysomes) during this period of time.

A fate for some of the UPR gene transcripts could be temporary sequestration in stress granules (SGs). SGs are membraneless aggregates of mRNAs and a variety of other proteins depending on the tissue and developmental stages in which the granules form (Buchan, Yoon, & Parker, 2011; Kosmacz et al., 2019; Wallace et al., 2015). Prominent among the proteins in SGs are RNA‐binding proteins such a polyadenylate(poly A)‐binding protein, a factor that binds to the poly A tails of mRNAs (Anderson & Kedersha, 2006; Chantarachot & Bailey‐Serres, 2018; Kedersha, Gupta, Li, Miller, & Anderson, 1999; Kosmacz et al., 2019). SGs are thought to be formed from mRNAs that are stalled in translation initiation and, therefore, can be found in association with translation initiation factors (Protter & Parker, 2016). SGs are dynamic structures and the mRNAs in the SGs are thought to enter the translation pool once released (Decker & Parker, 2012). SG formation in plants and other organisms has been observed in response to stresses, such as heat, hypoxia, starvation, treatment with metabolic inhibitors, or darkness (Cherkasov et al., 2013; Farny, Kedersha, & Silver, 2009; Jain et al., 2016; Kosmacz et al., 2019; Kroschwald et al., 2015; Sorenson & Bailey‐Serres, 2014). However, accumulation of SGs has not been demonstrated in response to ER stress in plants.

Here, we use ribosome profiling (Ingolia, Ghaemmaghami, Newman, & Weissman, 2009; Juntawong, Hummel, Bazin, & Bailey‐Serres, 2015) and polysome analyses to determine whether there are general translation changes in response to ER stress in maize seedlings. We find that, despite a burst of UPR gene transcription upon ER stress treatment, there is little change in translation. As a result, translation efficiency declines on a per RNA basis suggesting that some of the transcripts are not engaged by the translational machinery. We provide evidence that many of the UPR gene transcripts are not loaded onto polysomes, but they accumulate in SGs instead.

## 2 MATERIALS AND METHODS

### 2.1 Plant material

Tunicamycin treatment was performed largely as in Srivastava et al. (2018). In brief, sterile maize B73 seeds were placed in sterile Sigma bottles (Cat No V8630‐E100) with two layers of wet filter paper. The seeds were incubated at 30°C for 2 days then transferred to an illuminated incubator at 23°C for 7 days. Seedlings were treated with 5 µg/ml TM in DMSO for 0, 6, 12 hr and 12 hr mock treated.

### 2.2 RT‐PCR and qRT‐PCR analysis

Total RNA of maize seedling roots was extracted using RNeasy Plant mini kit (Qiagen) according to the manufacturer's instructions. RNA loaded onto polysomes (fractions 17‐24) was extracted using TRIzol (Invitrogen). Isolated RNA was reverse transcribed using Maxima H Minus First Strand cDNA Synthesis Kit (Thermo Scientific). For qPCR, 10‐fold (polysome RNA) or 20‐fold (total RNA) diluted cDNA, 10 pmol primer, and iQ SYBR Green Supermix (Bio‐Rad) were used for amplication in an iCycler IQ system (Bio‐Rad Laboratories). The qPCR data were normalized using tubulin (Zm00001d013367) as a standard for the polysome RNA analysis and ubiquitin (LOC100192952) for SG RNA analysis.

### 2.3 Polysome profiling

Polysomes were extracted from root tissue (0.3 g), which was flash frozen, ground in a mortar and pestle with liquid N2, and thawed in polysome extraction buffer (Chotewutmontri, Stiffler, Watkins, & Barkan, 2018) with modifications (50 mM Tris‐acetate (pH 8), 0.2 M KCl, 15 mM MgCl_2_, 0.2 M sucrose, 2 µg/ml pepstatin, 1 tablet/10 ml protease inhibitor, 2% polyoxyethylene‐10‐tridecyl ether, 1% Triton X‐100, 20 mM β‐mercaptoethanol, 3 mM DTT, 0.5 mg/ml heparin, 100 µg/ml chloramphenicol, 100 µg/ml cycloheximide). The homogenate was passed through a 40 µ filter followed by centrifugation at 4,700 *g* for 1 min at 4°C. The supernatant was collected and centrifuged at 21k *g* for 5 min at 4°C and centrifugation was repeated twice. The polysome extract was either used for fractionation immediately or flash frozen and stored at −80°C. The extract was layered onto 25%–65% sucrose gradient (50 mM Tris acetate (pH 8), 50 mM KCl, 15 mM MgCl2, 100 μg/ml cycloheximide, 100 μg/mL chloramphenicol) and centrifuged in a SW41 rotor (Beckman Coulter) at 35k g, 4°C, for 9 hr. Polysomes were fractionated using a Piston Gradient Fractionator (BioComp) equipped with a Econo UV monitor (Bio‐Rad) according to the manufacturer's instructions. Data were acquired using WinDAQ software (DATAQ Instruments).

### 2.4 Ribsome profiling library preparation

#### 2.4.1 RNase1 digestion

Ribosome profiling protocols from others were used with minor modifications (Chotewutmontri et al., 2018; Chung et al., 2015; Hsu et al., 2016). About 250 mg of 10‐day‐old maize B73 seedling roots (in biological duplicates) were ground with liquid N_2_ in 2.5 ml of polysome extraction buffer (PEB; 50 mM Tris‐Acetate (pH 8), 200 mM KCl, 15 mM MgCl_2_, 1% (v/v) Triton X‐100, 2% (v/v) polyoxyethelene 10‐tridecylether, 200 mM sucrose, 100 µg/ml cycloheximide, 100 µg/ml chloramphenicol, 20 mM β‐mercaptoethanol). The crude lysate was filtered through a 40 µm cell strainer (Falcon 08‐771‐1) by centrifugation at 4,000 *g* for 2 min. The flow through supernatant was further clarified by centrifugation at 21k *g* for 15 min. 300 µl of clarified lysate were aliquoted for total RNA extraction. For RPF generation, the remaining lysate was adjusted to A_254_ = ~10 (Lysate—PEB) with PEB. About 600 µl of the adjusted lysate were digested with 15 µl RNase1 (Ambion AM2295) for 30 min at 28°C on a shaker with slow speed. To terminate RNase digestion, 15 µl SUPERase‐In (Ambion AM2696) were added. For each sample, two‐aliquots of 600 µl (technical reps) were used and pooled after ultracentrifugation. The RNAse‐digested lysate was layered on a precooled 330 µl sucrose cushion (1 M sucrose, 40 mM Tris‐acetate (pH 8), 100 mM KCl, 15 mM MgCl_2_, 100 µg/ml cycloheximide, 100 µg/ml chloramphenicol, 10 mM β‐mercaptoethanol) in ultracentrifuge tubes (Thermo Scientific #45237) and subjected to ultracentrifugation at 131k *g* (57,000 rpm) for 90 min at 4°C in a Sorvall mini ultracentrifuge (Discovery M150) with S150‐AT fixed angle rotor (Thermo Scientific 45582). The pellet was resuspended in 150 µl ribosome dissociation buffer (10 mM Tris‐HCl (pH 7.5), 10 mM EDTA (pH 8), 5 mM EGTA (pH 8), 100 mM NaCl).

#### 2.4.2 Preparation of RPFs

RNA was purified by TRIzol extraction method until the phase separations step followed by addition of an equal volume of ice‐cold 100% ethanol to the aqueous phase and further purification using Zymo RNA Clean & Concentrator™‐5 columns (Zymo R1016) according to the manufacturer's protocol and quantified using a Nanodrop spectrophotometer. Quality of RPFs was assessed by electrophoresis of denatured RNA in a 15% TBE‐Urea gel (Invitrogen EC6885BOX) at 120 V for 5 min and 200 V for 55 min, staining the gel with SYBR gold (Invitrogen S11494) and determining the sharpness of the RPF band between the 28 nt and 34 nt RNA size markers. If the RPF bands appeared sharp for all samples, the RNAs from two technical duplicates were pooled for each sample. Subsequently, 10 µg RNA was treated with 1 µl Turbo DNase (Ambion AM2238) in a 50 µl reaction with 1x Turbo DNase buffer at 37°C for 30 min followed by addition of 1 µl more Turbo DNase and incubation at 37°C for additional 30 min, purification by Zymo RNA clean & concentrator −5 columns (Zymo R1016) and quantification with a Nanodrop spectrophotometer. Quality of the DNase‐treated RNA was assessed as above. rRNA depletion was carried out using a half reaction of Ribo‐Zero for plant seed/root kit (Illumina MRZSR116) per ~5 to 10 µg of DNAse‐treated RNA sample followed by RNA clean up using Zymo RNA clean & concentrator −5 columns (Zymo R1016) according to the Illumina TruSeq Ribo Profile kit protocol (Illumina 15066016). RNA was quantified using a Qubit RNA HS kit (Invitrogen Q32852) yielding ~20% recovery from the input.

For size‐selecting the RPFs, denatured rRNA‐depleted RNA was subjected to electrophoresis on a 15% TBE‐Urea gel (Invitrogen EC6885BOX) at 120 V for 5 min and 170 V for 85 min, stained in the gel with SYBR gold (Invitrogen S11494), and visualized on a blue light transilluminator. The gel region between 28 nt and 34 nt RNA size markers was excised and transferred to a 0.5 ml tube with a hole at the bottom made by a 18 G syringe needle, and the tube was placed a 2 ml microfuge tube. The tube was then centrifuged at 21k *g* for 2 min at 4°C to crush the gel slice and transfer its contents to the 2 ml microfuge tube. About 500 µl of RNA gel extraction buffer (0.3 M NaOAc (pH5.5), 1 mM EDTA (pH 8), 10 mM Tris‐HCl (pH 7.5), 0.25% (w/v) SDS) was added and incubated overnight at 4°C on a shaker. Eluted RPFs were filtered through 0.22 µ SpinX cellulose acetate filter columns (Sigma‐Aldrich CL8161). About 2 µl of Glyco Blue (Ambion AM9516) and equal volume of ice‐cold 100% isopropanol were added to the supernatant, and RPFs were precipitated at −80°C for 3 hr followed by centrifugation for 45 min at 21k *g* at 4°C, washing with ice‐cold 80% ethanol, and resuspending the pellet in 3.5 µl water.

Prior to library preparation, RPF ends were repaired using T4PNK kit (Thermo Scientific EK0031) as follows: 3.25 µl RNA was incubated at 70°C for 3 min and transferred to ice, followed by addition of 0.5 µl 10x T4 PNK buffer A (no ATP), 0.25 µl superase‐IN, 0.5 µl T4PNK enzyme, incubation at 37°C for 30 min, addition of 0.5 µl 10 mM ATP, incubation at 37°C for 1 hr. Following the addition of 5.5 µl water, the reaction was incubated at 75°C for 10 min and transferred to ice. Subsequently, cDNA libraries were prepared using Nextflex small RNAseq kit v3 (Bioo Scientific 5132‐05) according to the manufacturer's protocol and 12 cycles of PCR. Quality of the libraries was assessed using an Agilent bioanalyzer high sensitivity DNA Assay kit. Libraries were quantified using Qubit dsDNA HS Assay kit (Invitrogen Q32854), diluted and pooled together with RNAseq cDNA libraries for sequencing at the Iowa State University DNA Sequencing Facility on an Illumina HiSeq 3000 instrument.

### 2.5 RNAseq library preparation

Total RNA was extracted from 300 µl aliquoted clarified lysate using a TRIzol extraction method until the phase separation step and was followed by adding an equal volume of ice‐cold 100% ethanol to the aqueous phase and further purification using Zymo RNA clean & concentrator −5 columns (Zymo R1016) according to the manufacturer's protocol and quantified using a Nanodrop spectrophotometer. Integrity of total RNA was assessed by electrophoresis followed by DNase treatment and integrity assessment of DNase‐treated total RNA for ribo‐seq samples. rRNA depletion was carried out using half reactions of Ribo‐Zero for plant seed/root kit (Illumina MRZSR116) per ~5 µg of DNAse‐treated total RNA sample followed by RNA clean up using Zymo RNA clean & concentrator −5 columns (Zymo R1016) according to the Illumina TruSeq Ribo Profile kit protocol (Illumina 15066016). Total RNA was subjected to random fragmentation by alkaline hydrolysis as follows: 10 µl total RNA (~1 µg) mixed with 10 µl 2x fragmentation buffer (2 mM EDTA (pH 8), 12 mM Na_2_CO_3_, 88 mM NaHCO_3_), incubated at 95°C in a thermocycler for 20 min. The reaction was terminated by addition of 280 µl stop solution (0.3 M NaOAc, 53.6 µg/ml Glyco Blue) and 750 µl ice‐cold 100% ethanol followed by precipitation at −80°C for 3 hr. RNA was precipitated by centrifugation at 21k *g* for 45 min at 4°C and washed with ice‐cold 80% ethanol and resuspended in water. Size‐selection was carried out as above by excising the gel region between 28 nt and 34 nt RNA size markers. RNA was purified, end repaired and cDNA libraries were prepared as above. Quality of the libraries was assessed using an Agilent bioanalyzer high sensitivity DNA Assay kit. Libraries were quantified using Qubit dsDNA HS Assay kit (Invitrogen Q32854), diluted and pooled together with ribo‐seq cDNA libraries according to the DNA sequencing facility requirements at Iowa State University.

Ribo‐seq and RNAseq pooled libraries were multiplexed and sequenced in two lanes using an Illumina HiSeq 3000 to yield single‐end 50 bp reads. Because of low depth of ribo‐seq sequences, samples were pooled and resequenced in additional two lanes using Illumina HiSeq 3000 to yield single‐end 50 bp reads.

### 2.6 Ribo‐seq and RNAseq analysis

The quality of raw sequencing reads was assessed using FastQC (http://www.bioinformatics.babraham.ac.uk/projects/fastqc/). Cutadapt 1.16 (http://journal.embnet.org/index.php/embnetjournal/article/view/200) was used to remove adapters from raw sequencing reads using the following parameters: “‐a TGGAATTCTCGGGTGCCAAGG—discard‐untrimmed—minimum‐length 23.” Adapter‐trimmed reads were further processed to trim the four random bases, that were added to both ends of ribo‐seq and RNAseq reads during library preparation, using cutadapt 1.16 with the following parameters: “‐u 4 ‐u ‐4.” Subsequently, ribo‐seq reads that were 27 nt to 32 nt in length and RNAseq reads from 25 nt to 40 nt were retained, and the rest were filtered out. Subsequently, noncoding RNA (rRNA, snoRNA and tRNA) was depleted by mapping the reads to the maize ncRNA reference file (ftp://ftp.gramene.org/pub/gramene/CURRENT_RELEASE/fasta/zea_mays/ncrna/) using bowtie 1.2 (https://genomebiology.biomedcentral.com/articles/10.1186/gb‐2009‐10‐3‐r25) with the following parameters: “‐n 0 ‐l 23” and retaining only the unmapped reads. ncRNA‐depleted reads were subsequently mapped to maize cDNA and CDS reference transcriptomes (ftp://ftp.gramene.org/pub/gramene/CURRENT_RELEASE/fasta/zea_mays) using bowtie 1.2 with following parameters: “‐l 23 ‐v 2 ‐m 20—best ‐k 1” and only aligned reads were retained. cDNA and CDS‐aligned files were converted from sam to bam, sorted, indexed, and read counts were extracted using samtools view, sort, index, and idxstats tools, respectively (Li et al., 2009). All transcriptome‐level read counts were consolidated as gene‐level read counts. Pearson correlation data were plotted from CDS‐aligned read counts using the tool corrplot (https://github.com/taiyun/corrplot) in R studio after discarding genes that have zero read count in all the samples. True RPF characteristics, that is, RPFs mapping predominately to CDSs and showing triplet periodicity, were assessed with the R package ribo‐seqR (Hardcastle, 2014) using cDNA‐aligned ribo‐seq and RNAseq reads.

Sorted and indexed alignment bam files were run through igvtools with zoom level “7,” window function “mean” and window size “10.” The tdf files generated were used to make read coverage plots using Integrated Genomics Viewer (Thorvaldsdottir, Robinson, & Mesirov, 2013). Statistical analyses were performed using CDS‐aligned ribo‐seq and RNAseq read counts as follows. For identification of genes with differential translation efficiency, the CDS‐aligned read counts data were normalized to the housekeeping gene, actin. We used a Poisson distribution to model RNAseq data, while a zero‐inflated Poisson distribution to model the ribo‐seq data for dealing with the excess of zeros in RPF samples. Then we adopted a hierarchical modeling framework to assess the change of translational efficiency between time points. The level 1 model is a generalized linear mixed model that simultaneously models the expression means of ribo‐seq and RNAseq data, with abundance level, time point, and change of translational efficiency between time points as fixed effects, pairing signal between RPF and mRNA samples as a random effect. The level 2 models are for each dependent variable involved in level 1 model. For example, the parameter of change of translational efficiency for each gene is modeled as a mixture of 1 (for those genes without change of translational efficiency) and a Gamma distribution (for those genes with change of translational efficiency). Non‐informative priors were utilized to represent all uncertainties within the model. Posterior inferences such as calculating the posterior mean of the change of translational efficiency, or testing whether the translational fold change falls in a certain interval (e.g., at least twofold) while controlling FDR, were implemented via Markov Chain Monte Carlo sampling scheme. Details on the statistical method we proposed are in Supplemental Methods 1. The histogram and scatterplots were generated using ggplot2 in R studio (Wickham, 2016).

### 2.7 GO term enrichment analysis

For identification of differentially expressed genes from total reads and RPF reads, edgeR (Robinson, McCarthy, & Smyth, 2010) was used with actin normalization and genes with more than twofold change and with Hochberg multiple correction of FDR <0.05 were retained. AgriGO version 2 (http://bioinfo.cau.edu.cn/agriGO/) was used for singular enrichment analysis using hypergeometric test, multitest adjustment by Hochberg FDR <0.05 and minimum number of mapping entries set as 3. Negative log10 of corrected p‐values was calculated and top ten GO terms in each of the three categories were plotted. The background used by AgriGO was locus ID v3.30 (Gramene release 50).

### 2.8 PAB2 cloning

Maize polyA binding protein2 (PAB2) was identified in a blast search of maize sequences from Maize GDB using the Arabidopsis PAB2 (At4g34110) sequence. The ORF of the maize PAB2 homolog, Zm00001d005276 (GRMZM2G102829) was amplified from cDNA and cloned at the AscI and XbaI sites of the vector pSKM36 (Liu, Srivastava, Che, & Howell, 2007). A YFP tag was inserted at the C‐terminus of PAB2 to generate ZmPAB2‐YFP. The DNA was sequenced and aligned for verification.

### 2.9 Protoplast preparation and treatment

Leaf mesophyll protoplasts were prepared from B73 maize seedlings as described by Sheen et al, (http://molbio.mgh.harvard.edu/sheenweb/protocols_reg.html). DNA was purified using the Plasmid Miniprep Kit from Sigma and adjusted at a concentration of 1 µg/µl for transfection into protoplasts. After transformation, the protoplasts were incubated overnight. TM (5 µg/ml) treatment was carried out the next morning for 3 and 6 hr. Untreated protoplasts and protoplasts TM treated for 3 and 6 hr were visualized for SGs using a Leica SP5X MP confocal laser scanning microscope with a 63X oil immersion objective lens and excitation and emission wavelengths set at 520 and 550 nm for YFP.

### 2.10 SG enrichment

Untreated and 6 hr TM‐treated protoplasts were collected by centrifugation and the pellets were frozen for SG isolation. SG purification was adapted from Wheeler, Jain, Khong, and Parker (2017). The protoplasts were resuspended in 1 ml SG lysis buffer. Lysis buffer was prepared fresh before lysing cells; 50 mM Tris‐HCl (pH 7.4), 100 mM KOAc, 2 mM MgOAc, 0.5 mM DTT, 50 µg/ml heparin, 0.5% NP40, complete mini EDTA‐free protease inhibitor (1 tablet/50 ml lysis buffer, Sigma‐Aldrich), 1 U/ml RNasein Plus RNase Inhibitor (N2615, Promega). The resuspended protoplasts were lysed by passage through a 25G needle seven times at 4°C. The lysate was transferred to a microcentrifuge tube, centrifuged at 1,000 *g* for 5 min at 4°C to remove cell debris. The supernatant was transferred to a new microcentrifuge tube and centrifuged at 18k *g* for 25 min at 4°C to enrich for SG cores in the pellet. The supernatant was discarded and the pellet was checked under a microscope for fluorescence. The pellet was resuspended in 1 ml SG lysis buffer and then, centrifuged again at 18k *g* for 25 min at 4°C. The pellet so obtained was resuspended in 300 µl SG lysis buffer and centrifuged at 850 *g* for 2 min at 4°C. The supernatant representing the SG core‐enriched fraction was transferred to a new microcentrifuge tube, and RNA was extracted from this fraction using TRIzol (Invitrogen) following the manufacturer's protocol.

### 2.11 SUnSET assays

SUnSET assays were carried out using published protocols with minor modifications (Van Hoewyk, 2016). Nine‐day‐old maize seedlings were treated with water (mock) or 5 µg/ml TM for 6 and 12 hr followed by treatment with 50 µM puromycin solution (Sigma‐Aldrich P8833‐25MG) or water (no puromycin negative control) for 30 min. Seedlings were rinsed with water and 200–300 mg of roots were ground with mortar and pestle in liquid nitrogen followed by addition of protein extraction buffer (20 mM Tris‐HCl (pH 7.5), 50 mM NaCl, 1 mM EDTA (pH 8), 0.1% (v/v) Triton X‐100, 10% (v/v) glycerol, 2 mM NaF, 1 mM PMSF, 5 mM DTT, 1 tablet of Mini Complete protease inhibitor cocktail (Roche 4693124001)). The crude lysate was vortexed, centrifuged at 21k *g* for 3 min at 4°C, and the supernatant was centrifuged again at 21k *g* for 5 min at 4°C followed by quantification using a Qubit protein assay kit (Invitrogen Q33212). About 20 µg of total protein was mixed with equal volume of 2x Laemmli buffer (with β‐mercaptoethanol; BioRad), incubated in boiling water for 5 min and loaded in 4%–15% Mini‐protean TGX stain‐free protein gel (BioRad). Electrophoresis was carried out in 10x Tris/glycine/SDS buffer (BioRad) at 100 V for 100 min followed by imaging for total protein as a loading control. Subsequently, proteins were transferred from gel to a PVDF membrane using Invitrogen iBlot2 instrument, the membrane was blocked with 5% nonfat milk at room temperature (RT) for 1 hr, washed with 1x TBST (Tris‐buffered saline with Tween 20) at room temperature for 5 min, incubated with anti‐puromycin primary antibody (DSHB, University of Iowa, PMY‐2A4) in 0.5% nonfat milk (antibody final concentration was 0.48 µg/ml) overnight at 4°C, washed three times with 1x TBST for 5 min, incubated with secondary antibody (anti‐mouse IgG from sheep‐conjugated with HRP) in 0.5% nonfat milk (3:10,000 dilution) at room temperature for 1 hr and washed three times with 1x TBST for 5 min. Images were developed using a SuperSignal West Pico Plus chemiluminescent kit (Thermo Scientific) according to the manufacturer's protocol.

## 3 RESULTS

### 3.1 Ribosome profiling to assess the level of engagement of UPR gene transcripts by the translational machinery

**FIGURE 2:**
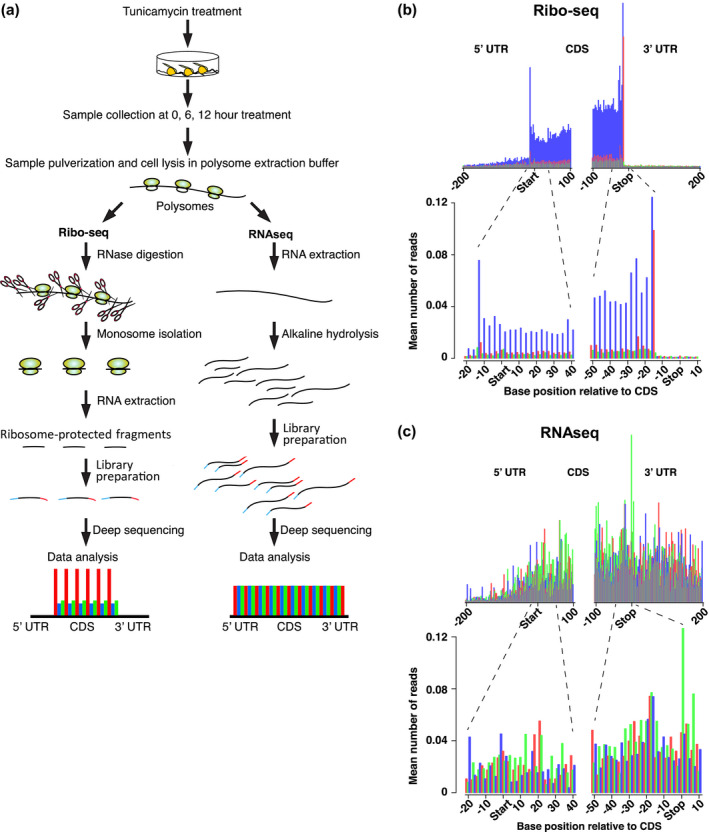
Use of riboprofiling to assess translation efficiency. (a) Ribosome profiling schematics shows the procedures for performing ribo‐seq and RNAseq analysis. (b) Metagene analysis conducted for quality control of the ribo‐seq analysis. Upper profile is a view of the number of reads in the 5′ and 3′ regions of the coding sequences (CDS) and the 5′ and 3′ UTRs for all genes in the analysis, and the lower profile is an expanded view of the 5′ and 3′ regions of the coding sequences. Profiles are plots of the mean number of ribosome protected fragments (RPFs) at various positions along expressed maize genes. RPFs exhibit a triplet periodicity reflecting the saltatory movement of ribosomes during translation. Bar colors correspond to the position in each codon to which the 5′ end of each RPF maps. Alignment of the 5′ ends of the 30 nt RPFs with respect to the base positions of the coding sequence (CDS). Higher peaks indicate ribosome pause sites. (c) Metagene analysis of the RNAseq data in which the mean number of total RNA reads are plotted at various positions along expressed maize genes. Alignment of the 5′ ends of the 30 bp cDNAs from the RNAseq analysis with respect to the base positions of the CDS. Neither the condensed (upper) nor the expanded (lower) profiles show, as expected, triplet periodicity as in the RPFs

The UPR is induced in maize seedlings in response to treatment with the ER stress agent, tunicamycin (TM) (Li, Humbert, & Howell, 2012). The induction of the UPR in maize is characterized by a burst in synthesis of mRNAs from the canonical UPR genes (Srivastava et al., 2018). To determine whether these mRNAs became actively engaged in the translational machinery, we conducted ribosome profiling (ribo‐seq) utilizing a modification of techniques developed previously for maize (Chotewutmontri et al., 2018). In our protocol, polysomes and total RNA were isolated in biological duplicates from maize seedling roots at 0, 6, and 12 hr after TM stress treatment. Polysomes were treated with RNase 1 to digest mRNA regions that were not protected by ribosomes, and the ribosome‐protected fragments (RPFs) were used to generate RPF cDNA libraries. Additionally, total RNA was isolated from the same tissues, fragmented by limited base hydrolysis, and used to generate cDNA libraries (Figure 2a).

From the sequencing data, we assessed the quality of the ribosome profiling reads based on the following criteria: (a) Size distributions of the sequenced reads in the RPF libraries were distributed around a mean of 32–34 nt with a shoulder at around 30 nt (Figure S1), similar to the sizes reported in Arabidopsis and maize (Chotewutmontri et al., 2018; Hsu et al., 2016; Juntawong et al., 2015). The 30 nt footprints in our study agree with those found previously in maize (Chotewutmontri et al., 2018), but are slightly larger than the 28 nt footprints observed in Arabidopsis (Hsu et al., 2016). The reads in the total RNA library were somewhat more broadly distributed but also centered around 31 nt. Thus, the two libraries were similar in terms of sizes of cDNA fragments. (b) Both ribo‐seq and RNAseq libraries showed a high degree of similarity between duplicates as assessed by Pearson correlation coefficient analysis (Figure S2). (c) Because ribo‐seq reads are derived from translating‐ribosome protected mRNA fragments, they map predominantly to the coding sequence and minimally to the UTRs (Figure 2b, upper profile). In contrast, the mapped RNAseq reads have a uniform distribution across the transcripts because they are derived from random fragmentation of total RNA (Figure 2c, upper profile). (d) During translation, ribosomes display saltatory movements, pausing momentarily at each codon. Therefore, upon metagene analysis, the 5′ ends of RPFs map at every third base in the CDS. This property is known as triplet periodicity and is shown exclusively by ribo‐seq data, while the RNAseq data do not show triplet periodicity. Indeed, triplet periodicity was observed in the metagene analysis of RPFs of 30 nt in length (Figure 2b, lower profile). The 30 nt reads from RNAseq data do not show triplet periodicity (Figure 2c, lower profile). (e) The 5′ ends of the RPFs mapped 13 nt upstream from the start codon in the P site of the ribosome, and 15 nt upstream from the stop codons, indicating that ribosomes disengage from the RNA when encountering a stop codon. These results are consistent with previous observations that about 30 nt of the mRNA are protected from nuclease by the 80S ribosome with about 15 nt between the first nuclease‐accessible nucleotide at the 5’ end of the RPF and the codon in the P site (Ingolia et al., 2009). (f) Another diagnostic feature of true RPFs is the out‐of‐frame peak 15 nt upstream of the stop codon (red bar, Figure 2b). The peak (tallest red bar) reveals that ribosome structure is altered upon release factor binding, allowing RNase 1 to digest the mRNA at a position one nucleotide off relative to that which occurs in mRNA on ribosomes during the elongation phase of translation (Alkalaeva, Pisarev, Frolova, Kisselev, & Pestova, 2006; Chung et al., 2015).

### 3.2 RNA sequencing and ribosome profiling data reveal a decrease in mRNA translation efficiencies in response to ER stress

**FIGURE 3:**
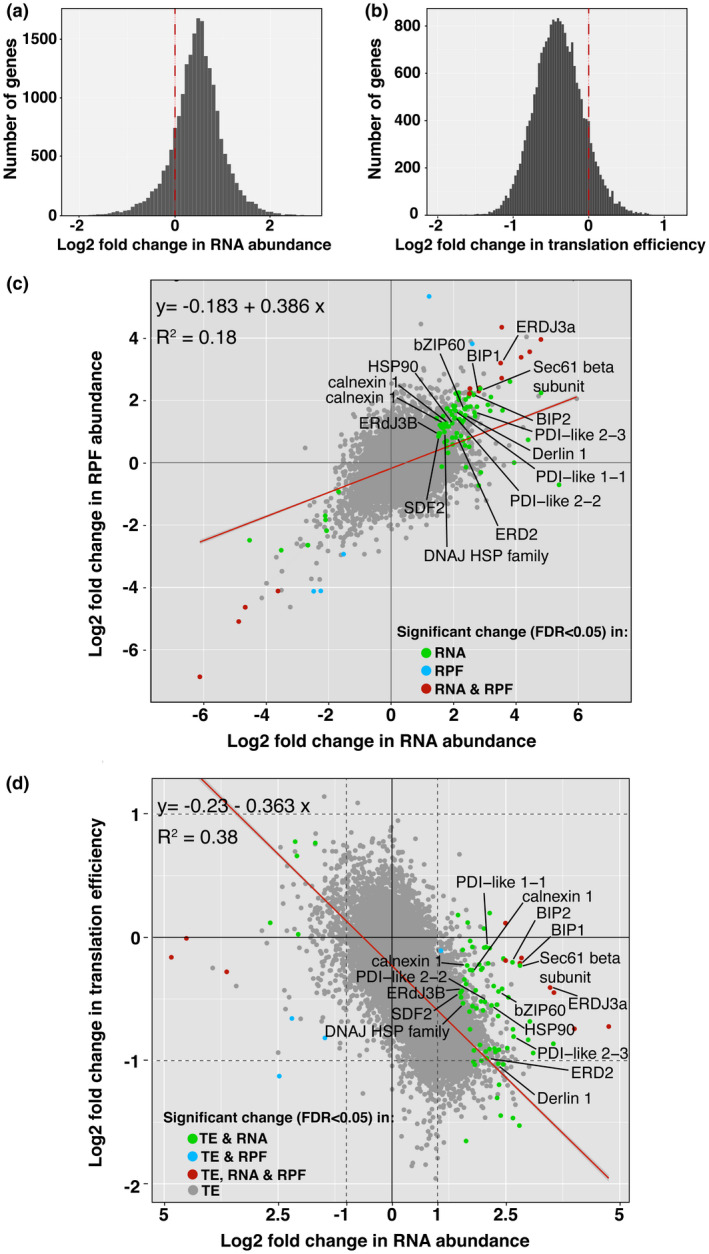
Change in RPF abundance and translation efficiency in response to ER stress. (a) Graph shows that the log_2_ fold change in RNA abundance for many genes increases at 12 hr post TM treatment compared to 0 hr. (b) Plot of log_2_ fold change in translation efficiency. Translation efficiency is the ratio of the abundance of RPFs to RNAs. (c) Scatterplot comparing the log_2_ fold change in RPF abundance versus the log_2_ fold change in RNA abundance for all the genes indicated with gray dots. UPR genes with significant changes in RNA abundance are highlighted with green dots. Red line is the regression line for which the coefficient of determination is shown. Other colored dots as indicated. (d) Scatterplot comparing the log_2_ fold change in translation efficiency versus the log_2_ fold change in mRNA abundance. Genes marked with green dots are canonical UPR genes with significant changes in translational efficiency and changes in RNA abundance. Many of these genes tend to have abundant RNAs, but are more downregulated in translation efficiency than the vast majority of other genes. Red line is the regression line for which the coefficient of determination (*R* ^2^) is shown. Other colored dots as indicated. ER, endoplasmic reticulum; UPR, unfolded protein response

We compared the ribo‐seq and RNAseq data to assess the level of translational control following TM treatment. To evaluate the efficiency of translation, the abundance of RPFs was compared to that of RNA transcripts obtained from RNAseq analysis of the same samples. Translation efficiency of a mRNA was expressed as the ratio of RPF read counts to RNAseq read counts (Ingolia et al., 2009) at a given timepoint. A generalized linear model was constructed for simultaneously modeling ribo‐seq and RNAseq data, and a hierarchical Bayesian approach was then applied to assess changes of translational efficiency between time points. The number of genes with significant change in translation efficiency (FDR < 0.05) was plotted against log_2_ fold change in RNA abundance (Figure 3a) and log_2_ fold change in translation efficiency (Figure 3b). We were particulary interested in whether there were changes in translation efficiency of the UPR gene transcripts because levels for many of these RNAs rose, reaching a peak at 12 hr following TM treatment (Srivastava et al., 2018). When comparing 12 hr to 0 hr, we observed a decline in translation efficiencies for a sizeable portion of the RNA transcript population (Figure 3b). The mean level of translation efficiency decline was less than twofold, but substantial considering that it is a change for many genes. Subsequently, edgeR was used with RNAseq and ribo‐seq read counts to identify differentially expressed genes (FDR < 0.05). When the log_2_ fold change in RPF abundance was plotted in a scatterplot against log_2_ fold change in RNA abundance it could be seen that there was an increase in a sizeable number of RPFs with increasing RNA abundance (Figure 3c). For many of the UPR genes the increase in RNA abundance exceeded the increase in abundance of RPFs. When the log_2_ fold change in translation efficiency was plotted in a scatterplot against the abundance of various mRNAs, there was a decline in translation efficiency of a sizeable number of genes with increasing RNA abundance (Figure 3d). Also, when the translation efficiencies for mRNAs of some of the canonical UPR genes were compared to the change in abundance of their RNAs, these genes showed a greater increase in RNA abundance compared to most genes, but a decline in translation efficiency (Figure 3d). For example, Derlin 1, a canonical UPR gene (Oda et al., 2006; Srivastava, Liu, Guo, Yin, & Howell, 2009), its mRNA level was upregulated more than fourfold between 0 and 12 hr, while its translation efficiency declined twofold. Similarly, for ERDJ3a, another UPR induced gene (Genereux et al., 2015; Srivastava et al., 2018), its steady state mRNA level increased more than eightfold, while its translation efficiency declined about 1.5‐fold. That trend held for many of the other UPR genes. Because the translation efficiency of these UPR genes declined, we interpret this to mean that there were more RNAs produced from these genes than were translated during this time frame.

### 3.3 No change in global translation rate was observed as assessed by polysome profiling and SUnSET assay

Because the translation efficiency of UPR genes declined, we interpret this to mean that there were more RNAs produced from these genes than loaded onto polysomes during this time frame. To validate this interpretation, other means for assessing translation activity, including polysome profiles and SUnSET assays, were utilized.

**FIGURE 4:**
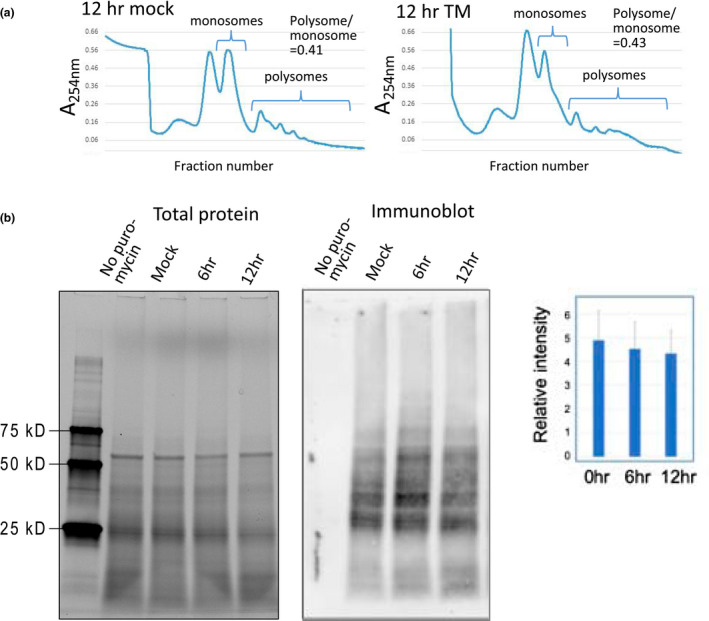
Assessments of rates of global protein synthesis. (a) Polysomes were profiled to ascertain whether there are global changes in the initiation rate of protein synthesis in the roots of TM treated seedlings. A decline in initiation rate would lead to a reduction in the ratio of polysomes to monosomes. Profiles from 25% to 65% sucrose density gradients show little difference between 12 hr mock and TM‐treated samples. Typical profiles are shown from over 10 gradients analyzed. (b) SUnSET assay to assess whether there are changes in rates of protein synthesis following TM treatment in seedlings. In this assay, protein synthesis is terminated and nascent proteins tagged with puromycin. Extracts from treated seedling roots are subjected to gel electrophoresis and immunoblotted with an antibody to puromycin. Bar graph shows the result of integrating the areas under the curves of densitometer scans for the different lanes of the immunoblots in five biological replicates of this experiment. Total protein bands are visualized by trihalo fluorophores in the gel that in the presence of UV light become covalently bound to the proteins, which can be visualized after transfer to membranes. Error bars are *SD*, *n* = 5

The polysome profiles on sucrose gradients have peaks showing ribosomal subunits, monosomes, followed by multiple peaks representing mRNAs with increasing numbers of translating ribosomes loaded on them. Changes in the shape of the overall profiles largely reflect changes in the global rate of initiation of protein synthesis, assuming that protein elongation rates are unchanged (Chasse, Boulben, Costache, Cormier, & Morales, 2017; Ingolia, Brar, Rouskin, McGeachy, & Weissman, 2012). For example, a global decline in protein synthesis initiation would appear in the polysome profile as an increase in monosomes and free subunits with a concomitant decline in polysomes. We did not observe much change in the polysome/monosome ratio in the polysome profile at 12 hr comparing the TM‐treated to untreated samples (Figure 4a). Therefore, we concluded that there was little, if any change at 12 hr in the overall initiation rate in response to ER stress.

As another measure of global translation in response to ER stress, we employed a surface sensing of translation (SUnSET) assay. The SUnSET assay utilizes puromycin to terminate translation and to tag the nascent proteins released from polysomes upon termination (Schmidt, Clavarino, Ceppi, & Pierre, 2009). Puromycin mimics the 3′ terminal nucleoside of a tRNA attached to an amino acid, but by a nonhydrolyzable amide bond. The amino acid mimic is incorporated by the ribosome onto the growing peptide chain, but it cannot be released from the nucleoside, causing chain termination. This assay, developed for animal cells, has been used successfully in plants (Van Hoewyk, 2016). Extracts containing the puromycin‐labeled proteins were subjected to gel electrophoresis, immunoblotted using an antibody against puromycin, and the immunoblot was scanned to determine the levels of protein synthesis (Figure 4b). No significant changes in global translation rates were detected in response to TM treatment. Thus, neither the rate of protein synthesis initiation nor global translation appear to change much in response to the UPR at the peak of UPR transcript accumulation.

### 3.4 mRNAs with levels that increase in response to UPR associate with SGs

**FIGURE 5:**
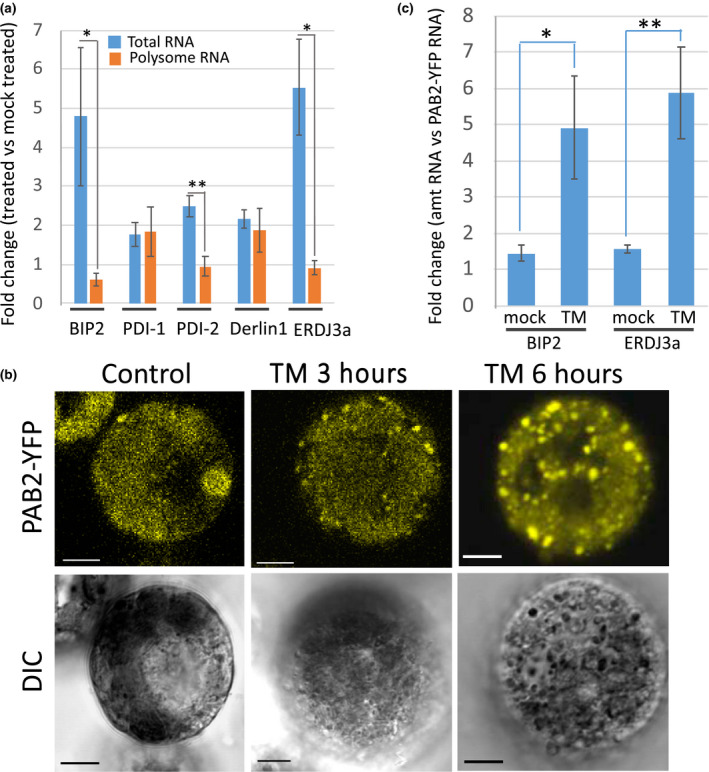
Disposition of UPR gene transcipts following ER stress treatment. (a) Presence of canonical UPR gene RNAs in total RNA and polysomes. Polysomes from seedling roots untreated or treated with TM for 12 hr were fractionated on sucrose density gradients. RNA was extracted from the polyribosome fractions and analyzed by qRT‐PCR. Data are presented as the fold change in RNA levels in fractions from TM‐treated versus mock‐treated plants. Bars represent the means of the fold changes from three biological reps. BIP2 (Zm00001d014993), PDI‐1 (Zm00001d04099), PDI‐2 (Zm00001d005866), Derlin 1 (Zm00001d010368), ERDJ3a (Zm00001d047726). Error bars = SEs. Asterisks indicate significance as determined by Student's *T* test. (b) Confocal images of maize leaf protoplasts transfected with the SG marker, PolyA binding protein 2‐YFP (PABP2‐YFP). Protoplasts were treated with TM and photographed at times indicated. DIC = Differential Interference Contrast microscopy. Bar = 10 µ. (c) Canonical UPR gene RNAs found in SG‐enriched fractions from untreated and 6 hr TM‐treated protoplasts. qRT‐PCR analysis of RNA extracted from triplicated SG‐enriched fraction samples. The cDNAs synthesized from the extracted RNA were spiked with equal amounts of recombinant PAB2‐YFP in order to compare RNA amounts in the treated and untreated samples. The qRT‐PCR results were expressed in terms of fold change (FC) over PAB2‐YFP mRNA. Error bars = SE. Asterisks indicate significance as determined by Student's *t* test. *Represents *p* < .05 and **represents *p* < .01. ER, endoplasmic reticulum; SG, stress granule; UPR, unfolded protein response

Given the decline in translation efficiency for the RNA transcripts for some of the canonical UPR genes, we asked what is the fate of these transcripts? To determine if some of the canonical UPR gene mRNAs were loaded onto polysomes, we obtained polysome fractions at 12 hr after TM treatment and compared the abundance of UPR RNAs on polysomes from TM‐treated samples to mock‐treated samples (Figure 5a). We found that the RNA abundance for some of the prominent UPR genes, BIP2, PDI‐2 and ERdJ3a, increased 2 to 5‐fold in total RNA fractions following TM treatment. However, the abundance of the mRNAs for these genes in the polysome fractions either held steady following TM treatment or declined somewhat. Thus, some of the UPR mRNAs were not loaded onto polysomes in proportion to their increase in abundance following TM treatment. Derlin1 represents an apparent exception which showed a decline in translation efficiency (Figure 3d) but little difference between its presence in total and polysome RNA at the 12 hr time point (Figure 5a). The discrepancy may be due to the fact that the former is a time course comparing 12 hr to 0 hr, whereas the latter is a snapshot in time (12 hr only).

We next determined how these mRNAs were being sequestered or stored. Structures well recognized for sequestering and storing mRNAs are SGs (Protter & Parker, 2016). SG formation due to ER stress has not been reported in plants, therefore, we used a poly(A)‐binding protein marker (PAB2‐YFP), that has been used by others, to visualize SGs in plants (Chantarachot & Bailey‐Serres, 2018). We transfected maize protoplasts with the SG marker and treated the protoplasts with TM. Fluorescent granules averaging about 0.5–1 micron in diameter, which increased in number with duration of treatment, became clearly visible (Figure 5b). (Note that the induction of UPR is more rapid in protoplasts than in seedlings as seen by the upregulation of bZIP60s, BIP2 and calnexin (CNX) at 6 hr (Figure S3)).

We investigated whether these granules contain UPR gene mRNAs, such as BIP2 and ERdJ3a mRNAs, by extracting RNA from fractions enriched in SGs. SG‐enriched fractions were obtained through a modification of a procedure developed by Wheeler et al. (2017) for the purification of SGs from mammalian cells. The procedure we developed for the purification of SGs from transfected, TM‐treated protoplasts involved similar centrifugation steps, enriching for the recovery of the PAB2‐YFP. (To compare the fractions from untreated and TM‐treated cells, we repeated the purification procedure without the aid of the PAB2‐YFP marker. We then spiked both fractions with recombinant PAB2‐YFP RNA and expressed the RNA levels as fold change with respect to PAB2‐YFP RNA.) We found that the SG‐enriched fraction from the 6 hr TM‐treated sample contained RNA transcripts from some of the UPR genes, BIP2 and ERdJ3a (Figure 5c). We also observed that the abundance of BIP2 and ERdJ3a transcripts in the SG fraction increased at 6 hr following TM treatment. We conclude that some RNA transcripts upregulated in the UPR are sequestered in SGs in an abundance comparable to their accumulation as total RNA.

**FIGURE 6:**
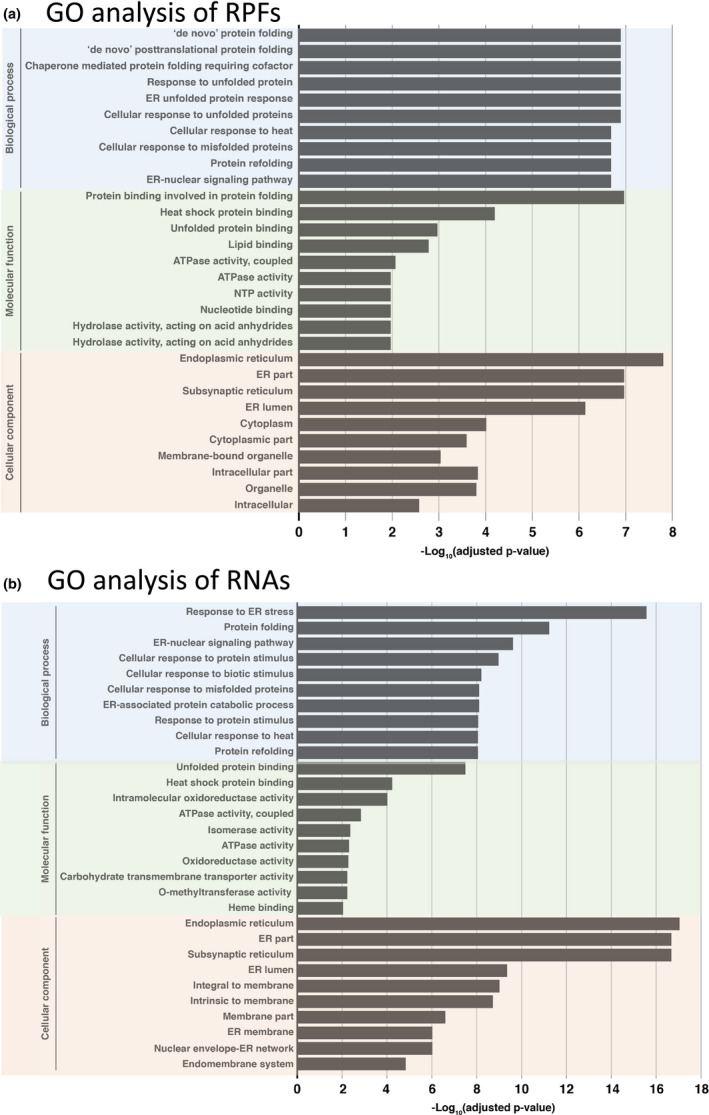
Gene ontology (GO) analysis of the RNAs and RPFs following TM treatment. (a) GO analysis of RPFs and (b) total RNA at 12 hr after TM treatment. GO analysis was conducted using AgriGO (http://bioinfo.cau.edu.cn/agriGO/) for the categories biological process, molecular function, and cellular compent

In studies of translation attenuation in response to hypoxic stress, a bias was found in the RNAs which were not sequestered in SGs but which were translated is spite of stress condtions (Sorenson & Bailey‐Serres, 2014). During hypoxic stress, the RNAs which remained in the translation pool during hypoxic stress were enriched in functions involving anaerobic metabolism. In our study, we conducted a GO term enrichment analysis of the differentially expressed genes in TM‐induced‐ER stress, that were identified using RPF reads and total RNA reads and found that GO terms from RPFs were enriched in biological process and molecular function for various aspects of protein folding (Figure 6a), similar to that found in total RNA (Figure 6b). Therefore, maize does not appear to selectively translate mRNAs, but seems to do so in proportion to their abundance in total RNA in response to ER stress. Even though there is no preferential translation of UPR mRNAs following stress treatment, the upsurge in their mRNA levels enables greater translation of the UPR genes relative to others.

## 4 DISCUSSION

Translation initiation is attenuated in mammalian cells in response to ER stress (Harding, Novoa, et al., 2000). It has been reasoned that attenuation prevents the further piling up of misfolded proteins in the ER when the demand exceeds the capacity for folding. The question we have addressed is whether translation is attenuated in plant cells in response to ER stress, and, if so, is it controlled in the same way as it is in mammalian cells. The answer is that translation is affected by ER stress in plants, but not in the same way as in mammalian cells.

Considering that plants lack a PERK homolog, plants cannot use a comparable PERK‐eIF2α‐P pathway for attenuating translation. Nonetheless, eIF2α is phosphorylated in Arabidopsis in response to various stresses (Zhang et al., 2008). However, eIF2α phosphorylation is not PERK dependent, but is GCN2 dependent (Zhang et al., 2008). Izquierdo et al. (2018) found that treatment of Arabidopsis with the ER stress agent, dithiothreitol (DTT), also induces the phosphorylation of eIF2α and that the phosphorylation is GCN1 and GCN2 dependent. Surprisingly, they found that DTT treatment attenuates protein synthesis as assessed by ^35^S‐Cys/Met incorporation, however the attenuation was not GCN1 and GCN2 dependent and, by inference not eIF2α dependent or UPR dependent. In any case, it has not been shown that eIF2α phosphorylation attenuates protein synthesis in plants. Izquierdo et al. (2018) and others have implied a limited role of eIF2α‐P in inhibiting protein synthesis in plants (Browning & Bailey‐Serres, 2015; Shaikhin, Smailov, Lee, Kozhanov, & Iskakov, 1992). As for the effect of DTT on protein synthesis, DTT is a strong reducing agent and is likely to affect translation unrelated to its effects on UPR. It is for this reason that we used TM to induce the UPR in this study and in other studies to determine the effects of persistent ER stress in plants (Srivastava et al., 2018).

**FIGURE 7:**
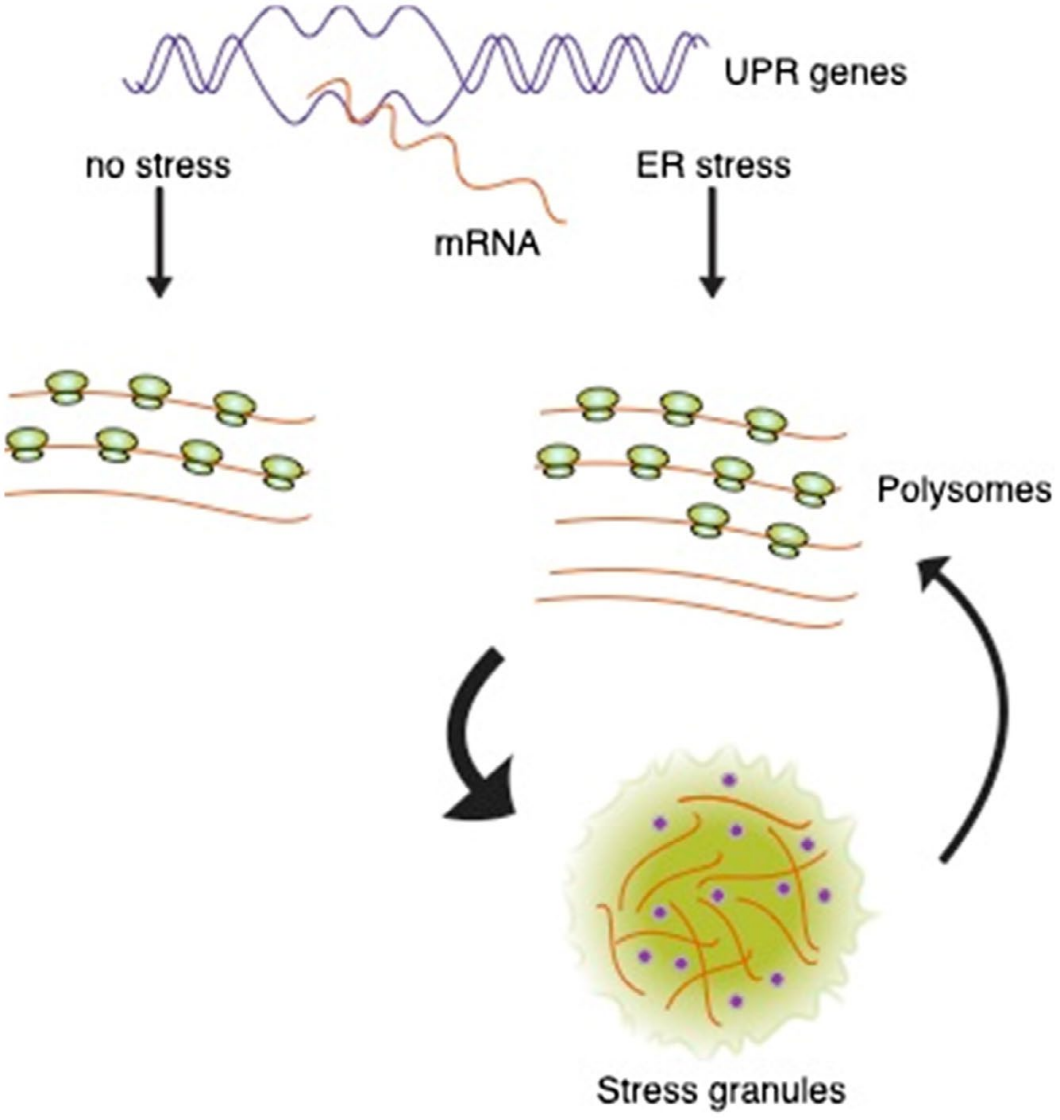
Model for the fate of RNA transcripts in response to ER stress. In response to ER stress, there is a surge in transcription which leads to an accumulation of transcripts from UPR genes, not all of which are immediately loaded onto polysomes. The untranslated RNAs drive the formation of stress granules which sequester the RNAs and other RNA‐binding proteins (blue dots). With time and dissolution of stress granules, the sequestered RNAs may be progressively liberated to enter the translation pool. ER, endoplasmic reticulum; UPR, unfolded protein response

In our hands, TM‐induced ER stress had little effect on the rate of protein synthesis initiation or global translation as assessed by polysome analysis and SUnSET assays. Although the rate of translation was very little affected, there was a reduction in translation efficiency for many mRNAs as assessed by ribosome profiling. The phenomenon is accompanied by a burst in transcription of UPR genes between 6 and 12 hr, during which time some of the transcripts are not loaded onto polysomes. We propose that some of these mRNAs are temporarily stored, presumably to enter the translational pool later (Figure 7).

We found that some of the mRNAs are sequestered in SG fractions. SGs are membraneless ribonucleoprotein bodies that consist of a stable core structure enveloped in a diffuse shell (Jain et al., 2016). The diffuse shell is thought to be a dynamic structure formed by liquid‐liquid phase separation events. SGs in mammalian cells are formed by or entrap mRNAs, the proteins that bind them, and other proteins, such as G3BP, a Ras‐GTPase‐activating protein SH3 domain‐binding protein that multimerizes in response to stress (Kedersha et al., 2016; Matsuki et al., 2013). SGs from mammalian cells have been isolated by immunoaffinity using G3BP (Khong et al., 2017), revealing that they have a diverse proteome as assessed by mass‐spec (Protter & Parker, 2016). SG proteomes have been analyzed in Arabidopsis and consist of many proteins also found in the SGs of human and yeast cells (Kosmacz et al., 2019). A number of the proteins found in Arabidopsis SGs belong together in protein networks that pre‐exist before stress treatment. To identify SGs in maize cells, we have used a fluorescent tagged poly(A)‐binding protein (PAB2‐YFP) which has been used as a marker for SGs in yeast (Brambilla, Martani, & Branduardi, 2017) and in other plants (Sorenson & Bailey‐Serres, 2014; Weber, Nover, & Fauth, 2008).

It is thought that messenger RNAs sequestered in SGs in mammalian cells are derived from the disassembly of polysomes as a consequence of translational repression brought about by ER stress (Protter & Parker, 2016). In fact, it is argued that activation of PERK and phosphorylation of eIF2α are required for SG formation during the UPR (Kimball, Horetsky, Ron, Jefferson, & Harding, 2003). Because translation initiation is stalled during ER stress in mammalian cells, SGs are thought to contain mRNAs in 48S preinitation complexes. In our case with maize, translation initation is not stalled and polysomes are not disassembled, yet SGs are formed in response to ER stress. We consider the force driving SG formation under these circumstances is the increased concentration of UPR gene transcripts produced during the burst in their synthesis in response to TM treatment. Since SGs are membraneless structures formed by perturbations that alter liquid‐liquid phase separations (Wheeler, Matheny, Jain, Abrisch, & Parker, 2016), it is possible that the surge in UPR gene transcription may promote the demixing phase transition that partitions ribonuclear protein complexes into physically discrete cytoplasmic structures such as SGs. The phase transitions that lead to SG formation are concentration dependent, and it could be that the macromolecular crowding of RNAs derived from the surge in RNA synthesis nucleates SG formation (Kedersha, Ivanov, & Anderson, 2013).

It is curious that in maize and mammalian cells, some UPR gene transcripts are sequestered and not all put to work immediately by the translation machinery. In mammalian cells, the translation machinery slows down to prevent the pile up of misfolded protein in the ER (Harding, Calfon, Urano, Novoa, & Ron, 2002; Harding, Novoa, et al., 2000). But it is the same machinery that is needed to produce the proteins, such as chaperones and other protein folding and quality control factors, needed to maintain homeostasis. In maize cells the translation machinery does not slow down nor does it speed up in response to ER stress, and some of the mRNAs needed to mitigate stress damage are put into reserve in the form of SGs.

In conclusion, we did not find significant changes in protein synthesis initiation in response to ER stress in maize seedlings. That might have been expected since plants have no known PERK homolog, and the attenuation of protein synthesis initiation in mammalian cells is largely due to the action of PERK in phosphorylating and inactivating the translation initiation factor eIF2α. However, in response to ER stress in maize there is a surge in expression of a constellation of canonical UPR genes (Srivastava et al., 2018). While these RNAs increase in numbers following ER stress treatment, we found using riboprofiling that their translation efficiency declines because many of the transcripts are not immediately engaged in the translation machinery, but are sequestered in SGs. This prevents the protein folding machinery in the ER from being overburdened by the translation of new transcripts from the UPR genes, and sequestration in SGs presumably provides a store for these important transcripts so that they can be rationed out as stress continues. Therefore, both plants and animals appear to have ways to prevent overburdening protein folding during ER stress by regulating translation.

## 5 ACCESSION NUMBERS

Maize version 4 locus identifiers

BIP2: Zm00001d014993

PDI‐1: protein disulphide isomerase‐like 1‐1(Zm00001d04099)

PDI‐2: protein disulphide isomerase 2‐3 (Zm00001d005866)

Derlin 1: Zm00001d010368

ERDJ3a: Zm00001d047726

Alpha tubulin 3 (Zm00001d013367)

Ubiquitin (LOC100192952)

Actin2: Zm00001d013873

Sequence data from this article can be found in NCBI’s Gene Expression Ominibus databases under record number GSE153969.

## CONFLICT OF INTEREST

The authors declare no conflict of interest associated with the work described in this manuscript.

## AUTHOR CONTRIBUTIONS

P.K, W.A.M., and S.H.H. conceived the experiments. P.K. conducted most of the experimental work. V.P. produced the polysomes profiling pattern and analyzed the RNAs in polysomes. R.S. demonstrated the presence of stress granules and analyzed the RNA content in the granules. R.B, P.K., and P.L performed the statistical analyses on the results.

## Supporting information

Fig S1‐S3‐Table S1

Supplementary Methods

Supplementary Material

## References

[pld3241-bib-0001] Alkalaeva, E. Z. , Pisarev, A. V. , Frolova, L. Y. , Kisselev, L. L. , & Pestova, T. V. (2006). In vitro reconstitution of eukaryotic translation reveals cooperativity between release factors eRF1 and eRF3. Cell, 125, 1125–1136. 16777602 10.1016/j.cell.2006.04.035

[pld3241-bib-0002] Anderson, P. , & Kedersha, N. (2006). RNA granules. The Journal of Cell Biology, 172, 803–808. 16520386 10.1083/jcb.200512082 PMC2063724

[pld3241-bib-0003] Brambilla, M. , Martani, F. , & Branduardi, P. (2017). The recruitment of the Saccharomyces cerevisiae poly(A)‐binding protein into stress granules: New insights into the contribution of the different protein domains. FEMS Yeast Research, 17 10.1093/femsyr/fox059 28873979

[pld3241-bib-0004] Browning, K. S. , & Bailey‐Serres, J. (2015). Mechanism of cytoplasmic mRNA translation. The Arabidopsis Book, 13, e0176. 26019692 10.1199/tab.0176 PMC4441251

[pld3241-bib-0005] Buchan, J. R. , Yoon, J. H. , & Parker, R. (2011). Stress‐specific composition, assembly and kinetics of stress granules in Saccharomyces cerevisiae. Journal of Cell Science, 124, 228–239. 21172806 10.1242/jcs.078444 PMC3010191

[pld3241-bib-0006] Chantarachot, T. , & Bailey‐Serres, J. (2018). Polysomes, stress granules, and processing bodies: A dynamic triumvirate controlling cytoplasmic mRNA fate and function. Plant Physiology, 176, 254–269. 29158329 10.1104/pp.17.01468 PMC5761823

[pld3241-bib-0007] Chasse, H. , Boulben, S. , Costache, V. , Cormier, P. , & Morales, J. (2017). Analysis of translation using polysome profiling. Nucleic Acids Research, 45, e15. 28180329 10.1093/nar/gkw907 PMC5388431

[pld3241-bib-0008] Cherkasov, V. , Hofmann, S. , Druffel‐Augustin, S. , Mogk, A. , Tyedmers, J. , Stoecklin, G. , & Bukau, B. (2013). Coordination of translational control and protein homeostasis during severe heat stress. Current Biology, 23, 2452–2462. 24291094 10.1016/j.cub.2013.09.058

[pld3241-bib-0009] Chotewutmontri, P. , Stiffler, N. , Watkins, K. P. , & Barkan, A. (2018). Ribosome profiling in maize. Methods in Molecular Biology, 1676, 165–183. 28986910 10.1007/978-1-4939-7315-6_10

[pld3241-bib-0010] Chung, B. Y. , Hardcastle, T. J. , Jones, J. D. , Irigoyen, N. , Firth, A. E. , Baulcombe, D. C. , & Brierley, I. (2015). The use of duplex‐specific nuclease in ribosome profiling and a user‐friendly software package for Ribo‐seq data analysis. RNA, 21, 1731–1745. 26286745 10.1261/rna.052548.115 PMC4574750

[pld3241-bib-0011] Clemens, M. J. (2001). Initiation factor eIF2 alpha phosphorylation in stress responses and apoptosis. Progress in Molecular and Subcellular Biology, 27, 57–89. 11575161 10.1007/978-3-662-09889-9_3

[pld3241-bib-0012] Decker, C. J. , & Parker, R. (2012). P‐bodies and stress granules: Possible roles in the control of translation and mRNA degradation. Cold Spring Harbor Perspectives in Biology, 4, a012286. 22763747 10.1101/cshperspect.a012286 PMC3428773

[pld3241-bib-0013] Deng, Y. , Humbert, S. , Liu, J. X. , Srivastava, R. , Rothstein, S. J. , & Howell, S. H. (2011). Heat induces the splicing by IRE1 of a mRNA encoding a transcription factor involved in the unfolded protein response in Arabidopsis. Proceedings of the National Academy of Sciences of the United States of America, 108, 7247–7252. 21482766 10.1073/pnas.1102117108 PMC3084119

[pld3241-bib-0014] Farny, N. G. , Kedersha, N. L. , & Silver, P. A. (2009). Metazoan stress granule assembly is mediated by P‐eIF2alpha‐dependent and ‐independent mechanisms. RNA, 15, 1814–1821. 19661161 10.1261/rna.1684009 PMC2743051

[pld3241-bib-0015] Gaddam, D. , Stevens, N. , & Hollien, J. (2013). Comparison of mRNA localization and regulation during endoplasmic reticulum stress in Drosophila cells. Molecular Biology of the Cell, 24, 14–20. 23135994 10.1091/mbc.E12-06-0491 PMC3530775

[pld3241-bib-0016] Genereux, J. C. , Qu, S. , Zhou, M. , Ryno, L. M. , Wang, S. , Shoulders, M. D. , … Wiseman, R. L. (2015). Unfolded protein response‐induced ERdj3 secretion links ER stress to extracellular proteostasis. The EMBO Journal, 34, 4–19. 25361606 10.15252/embj.201488896 PMC4291477

[pld3241-bib-0017] Hardcastle, T. J. (2014). riboSeqR: Analysis of sequencing data from ribosome profiling experiments. R package version 1.2.0.

[pld3241-bib-0018] Harding, H. P. , Calfon, M. , Urano, F. , Novoa, I. , & Ron, D. (2002). Transcriptional and translational control in the Mammalian unfolded protein response. Annual Review of Cell and Developmental Biology, 18, 575–599. 10.1146/annurev.cellbio.18.011402.160624 12142265

[pld3241-bib-0019] Harding, H. P. , Novoa, I. , Zhang, Y. , Zeng, H. , Wek, R. , Schapira, M. , & Ron, D. (2000). Regulated translation initiation controls stress‐induced gene expression in mammalian cells. Molecular Cell, 6, 1099–1108. 10.1016/S1097-2765(00)00108-8 11106749

[pld3241-bib-0020] Harding, H. P. , Zhang, Y. , Bertolotti, A. , Zeng, H. , & Ron, D. (2000). Perk is essential for translational regulation and cell survival during the unfolded protein response. Molecular Cell, 5, 897–904. 10.1016/S1097-2765(00)80330-5 10882126

[pld3241-bib-0021] Hollien, J. , & Weissman, J. S. (2006). Decay of endoplasmic reticulum‐localized mRNAs during the unfolded protein response. Science, 313, 104–107. 16825573 10.1126/science.1129631

[pld3241-bib-0022] Howell, S. H. (2013). ER stress responses in plants. Annual Review of Plant Biology, 64, 477–499. 10.1146/annurev-arplant-050312-120053 23330794

[pld3241-bib-0023] Hsu, P. Y. , Calviello, L. , Wu, H. L. , Li, F. W. , Rothfels, C. J. , Ohler, U. , & Benfey, P. N. (2016). Super‐resolution ribosome profiling reveals unannotated translation events in Arabidopsis. Proceedings of the National Academy of Sciences of the United States of America, 113, E7126–E7135. 27791167 10.1073/pnas.1614788113 PMC5111709

[pld3241-bib-0024] Ingolia, N. T. , Brar, G. A. , Rouskin, S. , McGeachy, A. M. , & Weissman, J. S. (2012). The ribosome profiling strategy for monitoring translation in vivo by deep sequencing of ribosome‐protected mRNA fragments. Nature Protocols, 7, 1534–1550. 22836135 10.1038/nprot.2012.086 PMC3535016

[pld3241-bib-0025] Ingolia, N. T. , Ghaemmaghami, S. , Newman, J. R. , & Weissman, J. S. (2009). Genome‐wide analysis in vivo of translation with nucleotide resolution using ribosome profiling. Science, 324, 218–223. 19213877 10.1126/science.1168978 PMC2746483

[pld3241-bib-0026] Iwata, Y. , & Koizumi, N. (2005). An Arabidopsis transcription factor, AtbZIP60, regulates the endoplasmic reticulum stress response in a manner unique to plants. Proceedings of the National Academy of Sciences of the United States of America, 102, 5280–5285. 15781873 10.1073/pnas.0408941102 PMC555978

[pld3241-bib-0027] Izquierdo, Y. , Kulasekaran, S. , Benito, P. , Lopez, B. , Marcos, R. , Cascon, T. , … Castresana, C. (2018). Arabidopsis nonresponding to oxylipins locus NOXY7 encodes a yeast GCN1 homolog that mediates noncanonical translation regulation and stress adaptation. Plant, Cell and Environment, 41, 1438–1452. 10.1111/pce.13182 29499090

[pld3241-bib-0028] Jain, S. , Wheeler, J. R. , Walters, R. W. , Agrawal, A. , Barsic, A. , & Parker, R. (2016). ATPase‐modulated stress granules contain a diverse proteome and substructure. Cell, 164, 487–498. 26777405 10.1016/j.cell.2015.12.038 PMC4733397

[pld3241-bib-0029] Juntawong, P. , Hummel, M. , Bazin, J. , & Bailey‐Serres, J. (2015). Ribosome profiling: A tool for quantitative evaluation of dynamics in mRNA translation. Methods in Molecular Biology, 1284, 139–173. 25757771 10.1007/978-1-4939-2444-8_7

[pld3241-bib-0030] Kadowaki, H. , & Nishitoh, H. (2019). Endoplasmic reticulum quality control by garbage disposal. The FEBS Journal, 286, 232–240. 29923316 10.1111/febs.14589

[pld3241-bib-0031] Kamauchi, S. , Nakatani, H. , Nakano, C. , & Urade, R. (2005). Gene expression in response to endoplasmic reticulum stress in Arabidopsis thaliana. The FEBS Journal, 272, 3461–3476. 15978049 10.1111/j.1742-4658.2005.04770.x

[pld3241-bib-0032] Kedersha, N. L. , Gupta, M. , Li, W. , Miller, I. , & Anderson, P. (1999). RNA‐binding proteins TIA‐1 and TIAR link the phosphorylation of eIF‐2 alpha to the assembly of mammalian stress granules. The Journal of Cell Biology, 147, 1431–1442. 10613902 10.1083/jcb.147.7.1431 PMC2174242

[pld3241-bib-0033] Kedersha, N. , Ivanov, P. , & Anderson, P. (2013). Stress granules and cell signaling: More than just a passing phase? Trends in Biochemical Sciences, 38, 494–506. 10.1016/j.tibs.2013.07.004 24029419 PMC3832949

[pld3241-bib-0034] Kedersha, N. , Panas, M. D. , Achorn, C. A. , Lyons, S. , Tisdale, S. , Hickman, T. , … Anderson, P. (2016). G3BP‐Caprin1‐USP10 complexes mediate stress granule condensation and associate with 40S subunits. The Journal of Cell Biology, 212, 845–860. 27022092 10.1083/jcb.201508028 PMC4810302

[pld3241-bib-0035] Khong, A. , Matheny, T. , Jain, S. , Mitchell, S. F. , Wheeler, J. R. , & Parker, R. (2017). The stress granule transcriptome reveals principles of mRNA accumulation in stress granules. Molecular Cell, 68, 808–820.e805. 29129640 10.1016/j.molcel.2017.10.015 PMC5728175

[pld3241-bib-0036] Kimball, S. R. , Horetsky, R. L. , Ron, D. , Jefferson, L. S. , & Harding, H. P. (2003). Mammalian stress granules represent sites of accumulation of stalled translation initiation complexes. American Journal of Physiology. Cell Physiology, 284, C273–284. 12388085 10.1152/ajpcell.00314.2002

[pld3241-bib-0037] Kosmacz, M. , Gorka, M. , Schmidt, S. , Luzarowski, M. , Moreno, J. C. , Szlachetko, J. , … Skirycz, A. (2019). Protein and metabolite composition of Arabidopsis stress granules. New Phytologist, 222, 1420–1433. 30664249 10.1111/nph.15690

[pld3241-bib-0038] Kroschwald, S. , Maharana, S. , Mateju, D. , Malinovska, L. , Nuske, E. , Poser, I. , … Alberti, S. (2015). Promiscuous interactions and protein disaggregases determine the material state of stress‐inducible RNP granules. Elife, 4, e06807. 26238190 10.7554/eLife.06807 PMC4522596

[pld3241-bib-0039] Lageix, S. , Lanet, E. , Pouch‐Pelissier, M. N. , Espagnol, M. C. , Robaglia, C. , Deragon, J. M. , & Pelissier, T. (2008). Arabidopsis eIF2alpha kinase GCN2 is essential for growth in stress conditions and is activated by wounding. BMC Plant Biology, 8, 134. 19108716 10.1186/1471-2229-8-134 PMC2639386

[pld3241-bib-0040] Li, H. , Handsaker, B. , Wysoker, A. , Fennell, T. , Ruan, J. , Homer, N. , … Genome Project Data Processing, S . (2009). The sequence alignment/map format and SAMtools. Bioinformatics, 25, 2078–2079. 19505943 10.1093/bioinformatics/btp352 PMC2723002

[pld3241-bib-0041] Li, Y. , Humbert, S. , & Howell, S. H. (2012). ZmbZIP60 mRNA is spliced in maize in response to ER stress. BMC Research Notes, 5, 144. 22417282 10.1186/1756-0500-5-144 PMC3369818

[pld3241-bib-0067] Liu, J. X. , Srivastava, R. , Che, P. , & Howell, S.H. (2007). An endoplasmic reticulum stress response in Arabidopsis is mediated by proteolytic processing and nuclear relocation of a membrane‐associated transcription factor, bZIP28. Plant Cell, 19, 4111–4119. 18156219 10.1105/tpc.106.050021 PMC2217655

[pld3241-bib-0042] Martinez, I. M. , & Chrispeels, M. J. (2003). Genomic analysis of the unfolded protein response in Arabidopsis shows its connection to important cellular processes. The Plant Cell, 15, 561–576. 12566592 10.1105/tpc.007609 PMC141221

[pld3241-bib-0043] Matsuki, H. , Takahashi, M. , Higuchi, M. , Makokha, G. N. , Oie, M. , & Fujii, M. (2013). Both G3BP1 and G3BP2 contribute to stress granule formation. Genes to Cells, 18, 135–146. 23279204 10.1111/gtc.12023

[pld3241-bib-0044] Mishiba, K. , Nagashima, Y. , Suzuki, E. , Hayashi, N. , Ogata, Y. , Shimada, Y. , & Koizumi, N. (2013). Defects in IRE1 enhance cell death and fail to degrade mRNAs encoding secretory pathway proteins in the Arabidopsis unfolded protein response. Proceedings of the National Academy of Sciences of the United States of America, 110, 5713–5718. 23509268 10.1073/pnas.1219047110 PMC3619347

[pld3241-bib-0045] Moore, K. , & Hollien, J. (2015). Ire1‐mediated decay in mammalian cells relies on mRNA sequence, structure, and translational status. Molecular Biology of the Cell, 26, 2873–2884. 26108623 10.1091/mbc.E15-02-0074 PMC4571326

[pld3241-bib-0046] Nagashima, Y. , Mishiba, K. , Suzuki, E. , Shimada, Y. , Iwata, Y. , & Koizumi, N. (2011). Arabidopsis IRE1 catalyses unconventional splicing of bZIP60 mRNA to produce the active transcription factor. Scientific Reports, 1, 29. 22355548 10.1038/srep00029 PMC3216516

[pld3241-bib-0047] Nakajima, Y. , & Suzuki, S. (2013). Environmental stresses induce misfolded protein aggregation in plant cells in a microtubule‐dependent manner. International Journal of Molecular Sciences, 14, 7771–7783. 23574938 10.3390/ijms14047771 PMC3645715

[pld3241-bib-0048] Oda, Y. , Okada, T. , Yoshida, H. , Kaufman, R. J. , Nagata, K. , & Mori, K. (2006). Derlin‐2 and Derlin‐3 are regulated by the mammalian unfolded protein response and are required for ER‐associated degradation. The Journal of Cell Biology, 172, 383–393. 16449189 10.1083/jcb.200507057 PMC2063648

[pld3241-bib-0049] Protter, D. S. W. , & Parker, R. (2016). Principles and properties of stress granules. Trends in Cell Biology, 26, 668–679. 27289443 10.1016/j.tcb.2016.05.004 PMC4993645

[pld3241-bib-0050] Robinson, M. D. , McCarthy, D. J. , & Smyth, G. K. (2010). edgeR: A Bioconductor package for differential expression analysis of digital gene expression data. Bioinformatics, 26, 139–140. 19910308 10.1093/bioinformatics/btp616 PMC2796818

[pld3241-bib-0051] Schmidt, E. K. , Clavarino, G. , Ceppi, M. , & Pierre, P. (2009). SUnSET, a nonradioactive method to monitor protein synthesis. Nature Methods, 6, 275–277. 19305406 10.1038/nmeth.1314

[pld3241-bib-0052] Shaikhin, S. M. , Smailov, S. K. , Lee, A. V. , Kozhanov, E. V. , & Iskakov, B. K. (1992). Interaction of wheat germ translation initiation factor 2 with GDP and GTP. Biochimie, 74, 447–454. 1637870 10.1016/0300-9084(92)90085-s

[pld3241-bib-0053] Sorenson, R. , & Bailey‐Serres, J. (2014). Selective mRNA sequestration by OLIGOURIDYLATE‐BINDING PROTEIN 1 contributes to translational control during hypoxia in Arabidopsis. Proceedings of the National Academy of Sciences of the United States of America, 111, 2373–2378. 24469793 10.1073/pnas.1314851111 PMC3926019

[pld3241-bib-0054] Srivastava, R. , Li, Z. , Russo, G. , Tang, J. , Bi, R. , Muppirala, U. , … Lawrence‐Dill, C. J. (2018). Response to persistent ER Stress in plants: A multiphasic process that transitions cells from prosurvival activities to cell death. The Plant Cell, 30, 1220–1242. 29802214 10.1105/tpc.18.00153 PMC6048783

[pld3241-bib-0055] Srivastava, R. , Liu, J. X. , Guo, H. , Yin, Y. , & Howell, S. H. (2009). Regulation and processing of a plant peptide hormone, AtRALF23, in Arabidopsis. The Plant Journal, 59, 930–939. 19473327 10.1111/j.1365-313X.2009.03926.x

[pld3241-bib-0056] Strasser, R. (2018). Protein quality control in the endoplasmic reticulum of plants. Annual Review of Plant Biology, 69, 147–172. 10.1146/annurev-arplant-042817-040331 PMC7039705 29570364

[pld3241-bib-0057] Thorvaldsdottir, H. , Robinson, J. T. , & Mesirov, J. P. (2013). Integrative Genomics Viewer (IGV): High‐performance genomics data visualization and exploration. Briefings in Bioinformatics, 14, 178–192. 22517427 10.1093/bib/bbs017 PMC3603213

[pld3241-bib-0058] Van Hoewyk, D. (2016). Use of the non‐radioactive SUnSET method to detect decreased protein synthesis in proteasome inhibited Arabidopsis roots. Plant Methods, 12, 20. 26989430 10.1186/s13007-016-0120-z PMC4794914

[pld3241-bib-0059] Wallace, E. W. , Kear‐Scott, J. L. , Pilipenko, E. V. , Schwartz, M. H. , Laskowski, P. R. , Rojek, A. E. , … Airoldi, E. M. (2015). Reversible, specific, active aggregates of endogenous proteins assemble upon heat stress. Cell, 162, 1286–1298. 26359986 10.1016/j.cell.2015.08.041 PMC4567705

[pld3241-bib-0060] Walter, P. , & Ron, D. (2011). The unfolded protein response: From stress pathway to homeostatic regulation. Science, 334, 1081–1086. 22116877 10.1126/science.1209038

[pld3241-bib-0061] Weber, C. , Nover, L. , & Fauth, M. (2008). Plant stress granules and mRNA processing bodies are distinct from heat stress granules. The Plant Journal, 56, 517–530. 18643965 10.1111/j.1365-313X.2008.03623.x

[pld3241-bib-0062] Wheeler, J. R. , Jain, S. , Khong, A. , & Parker, R. (2017). Isolation of yeast and mammalian stress granule cores. Methods, 126, 12–17. 28457979 10.1016/j.ymeth.2017.04.020 PMC5924690

[pld3241-bib-0063] Wheeler, J. R. , Matheny, T. , Jain, S. , Abrisch, R. , & Parker, R. (2016). Distinct stages in stress granule assembly and disassembly. eLife, 5, e18413. 27602576 10.7554/eLife.18413 PMC5014549

[pld3241-bib-0064] Wickham, H. (2016). ggplot2: Elegant graphics for data analysis. New York, NY: Springer‐Verlag ISBN 978‐3‐319‐24277‐4, Retrieved from https://ggplot2.tidyverse.org

[pld3241-bib-0065] Yan, W. , Frank, C. L. , Korth, M. J. , Sopher, B. L. , Novoa, I. , Ron, D. , & Katze, M. G. (2002). Control of PERK eIF2alpha kinase activity by the endoplasmic reticulum stress‐induced molecular chaperone P58IPK. Proceedings of the National Academy of Sciences of the United States of America, 99, 15920–15925. 12446838 10.1073/pnas.252341799 PMC138540

[pld3241-bib-0066] Zhang, Y. , Wang, Y. , Kanyuka, K. , Parry, M. A. , Powers, S. J. , & Halford, N. G. (2008). GCN2‐dependent phosphorylation of eukaryotic translation initiation factor‐2alpha in Arabidopsis. Journal of Experimental Botany, 59, 3131–3141. 18603615 10.1093/jxb/ern169 PMC2504353

