# Supplementary material for: Control of translation during the unfolded protein response in maize seedlings: Life without PERKs
Source: Plant Direct. 2020 Jul 30;4(7):e00241. doi: 10.1002/pld3.241 (PMC7390965; doi:10.1002/pld3.241)
Supplement: Supplementary file 1 — Fig S1‐S3‐Table S1 [file PLD3-4-e00241-s001.pptx]

## Slide 1
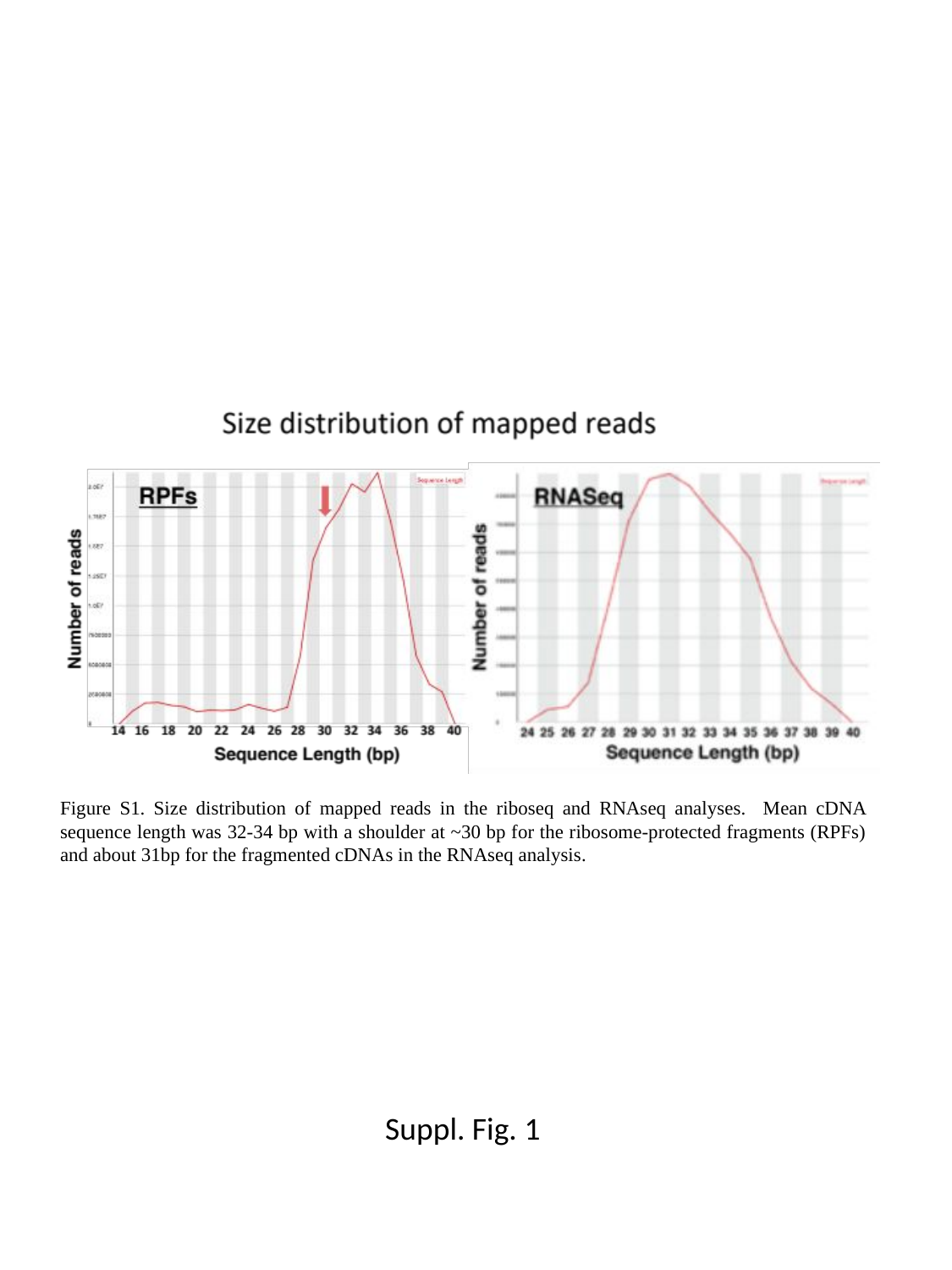

Figure S1. Size distribution of mapped reads in the riboseq and RNAseq analyses. Mean cDNA sequence length was 32-34 bp with a shoulder at ~30 bp for the ribosome-protected fragments (RPFs) and about 31bp for the fragmented cDNAs in the RNAseq analysis.
Suppl. Fig. 1

## Slide 2
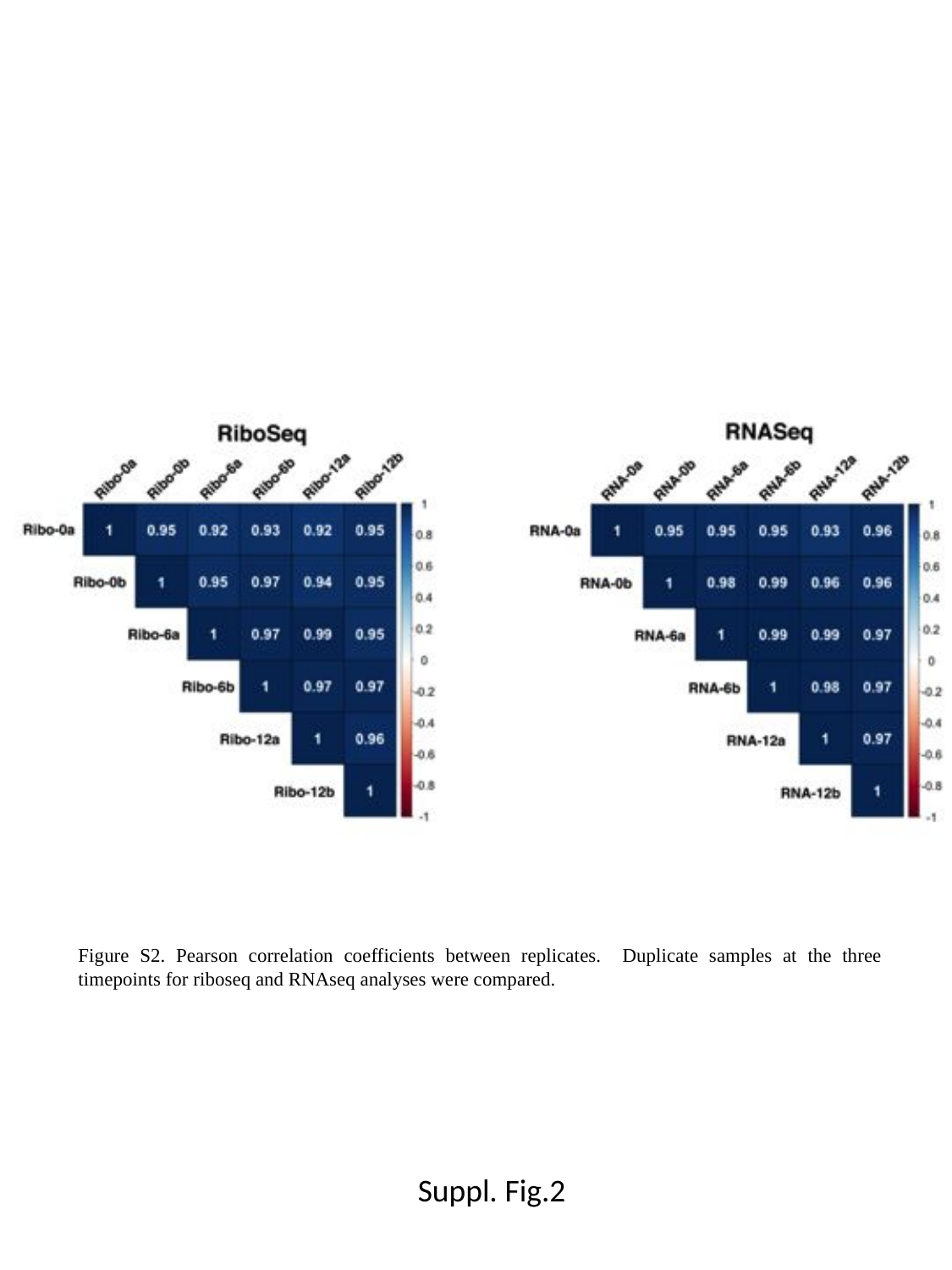

Pearson correlation coefficients between replicates
Figure S2. Pearson correlation coefficients between replicates. Duplicate samples at the three timepoints for riboseq and RNAseq analyses were compared.
Suppl. Fig.2

## Slide 3
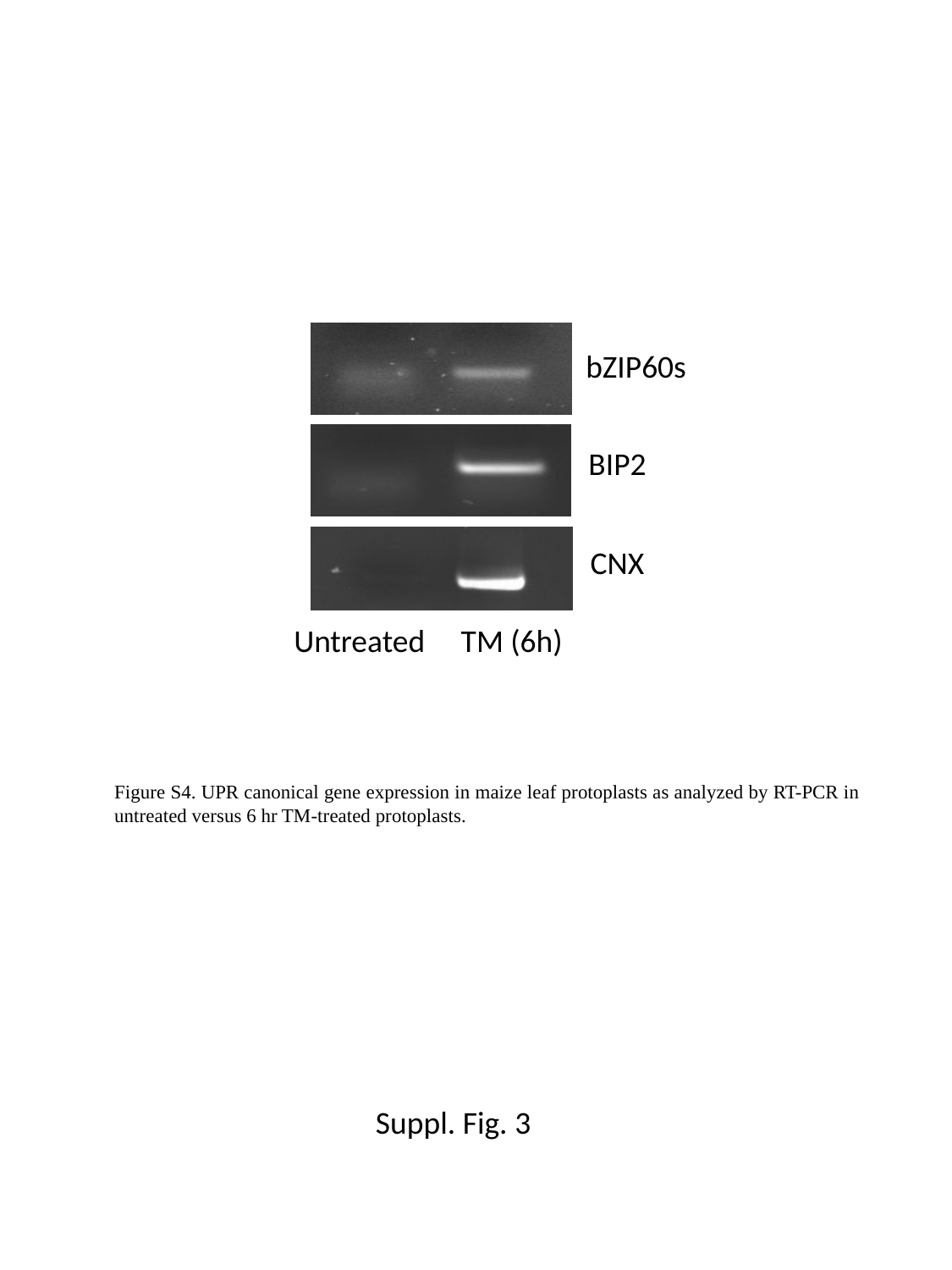

bZIP60s
BIP2
CNX
 Untreated TM (6h)
Figure S4. UPR canonical gene expression in maize leaf protoplasts as analyzed by RT-PCR in untreated versus 6 hr TM-treated protoplasts.
Suppl. Fig. 3

## Slide 4
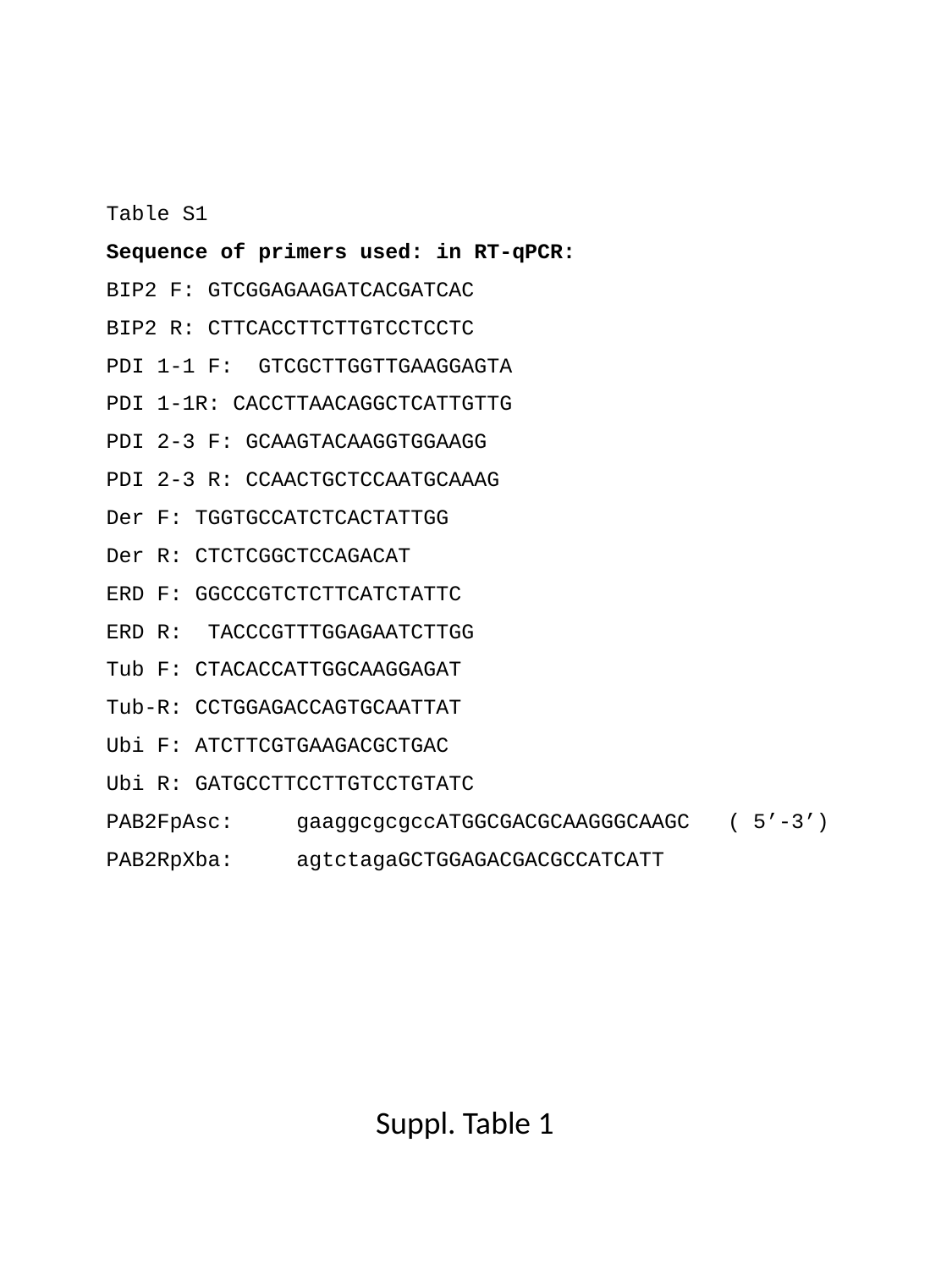

Table S1
Sequence of primers used: in RT-qPCR:
BIP2 F: GTCGGAGAAGATCACGATCAC
BIP2 R: CTTCACCTTCTTGTCCTCCTC
PDI 1-1 F:  GTCGCTTGGTTGAAGGAGTA
PDI 1-1R: CACCTTAACAGGCTCATTGTTG
PDI 2-3 F: GCAAGTACAAGGTGGAAGG
PDI 2-3 R: CCAACTGCTCCAATGCAAAG
Der F: TGGTGCCATCTCACTATTGG
Der R: CTCTCGGCTCCAGACAT
ERD F: GGCCCGTCTCTTCATCTATTC
ERD R:  TACCCGTTTGGAGAATCTTGG
Tub F: CTACACCATTGGCAAGGAGAT
Tub-R: CCTGGAGACCAGTGCAATTAT
Ubi F: ATCTTCGTGAAGACGCTGAC
Ubi R: GATGCCTTCCTTGTCCTGTATC
PAB2FpAsc: gaaggcgcgccATGGCGACGCAAGGGCAAGC ( 5’-3’)
PAB2RpXba: agtctagaGCTGGAGACGACGCCATCATT
Suppl. Table 1
