# Supplementary material for: Control of translation during the unfolded protein response in maize seedlings: Life without PERKs
Source: Plant Direct. 2020 Jul 30;4(7):e00241. doi: 10.1002/pld3.241 (PMC7390965; doi:10.1002/pld3.241)
Supplement: Supplementary file 2 — Supplementary Methods [file PLD3-4-e00241-s002.pdf]

Supplemental Methods file 1

Statistical Method

Suppose that the experiment measures G genes. Let Y_{gijk} denote read count data from gene g, i^{th} protocol, j^{th} time point, and k^{th} biological replicate, where $i = 1, 2$ correspond to RPF sample and mRNA sample respectively. Note that each biological replicate provides two samples, one generating ribo-seq measurements and the other generate whole transcriptome (mRNA-seq) measurements. Here we consider a comparison between two conditions, and assume n replicates in each condition, i.e., $j = 1, 2,\ k = 1, \ldots, n$.

Bayesian Modeling Pipeline

In the development of our modeling framework, we use zero-inflated Poisson distribution to model RPF count data Y_{g1jk}, for dealing with the excess of zeros . Let Z_{g1jk} be independently distributed with a binary probability mass function having parameter p_{g1jk}. Z_{g1jk} is used to specify which mixture component the observed count data Y_{g1jk} comes from. Take the Y_{g1jk}'s to be independent with conditional distributions given $Z_{g1jk} = 1$ that are Poisson distribution with parameter λ_{g1jk}. The distribution of Y_{g1jk} can be expressed as

$$\begin{aligned} Z_{g1jk}|p_{g1jk} &\overset{ind}{\sim} \text{Bernoulli}(p_{g1jk}) \\ Y_{g1jk}|Z_{g1jk}, \lambda_{g1jk} &\overset{ind}{\sim} \begin{cases} 0, & \text{if } Z_{g1jk} = 0 \\ \text{Poisson}(\lambda_{g1jk}), & \text{if } Z_{g1jk} = 1. \end{cases} \end{aligned} \tag{1}$$

The parameter p_{g1jk} is the probability of Y_{g1jk} coming from Poisson part, which may vary depending on the sequencing depth and gene g. Thus we consider a logistic relation modeled as follows,

$$logit(p_{g1jk}) = \beta_g \cdot S_{1jk}, \tag{2}$$

where S_{1jk} is the normalization factor that adjusts for sequencing depths variation and potentially other technical effects across the replicates.

For RNA-seq read count data Y_{g2jk}, we assume it follows a regular Poisson distribution with true expression mean parameter λ_{g2jk}, i.e.,

$$Y_{g2jk} \sim \text{Poisson}(\lambda_{g2jk}).$$

For both mRNA and RPF samples, λ_{gijk} represents the expression mean of kth replicate of jth time point in i^{th} protocol of gene g. We can express expectations on read counts λ_{gijk} as a function of normalization factor S_{ijk}, baseline quantity related to RPF abundance λ_g in the first time point, a quantity u_{gi} that relates the protocol difference between mRNA and RPF, a quantity t_{gj} that relates the fold change between two time points, a quantity w_{gij} that captures the effect of the time on translation, and a quantity b_{gjk} that relates to the pairing signal between RNA and RPF. In particular, the expected expression mean λ_{gijk} is modeled as below,

$$\lambda_{gijk} = S_{ijk} \cdot \lambda_g \cdot u_{gi} \cdot t_{gj} \cdot w_{gij} \cdot b_{gjk}, \tag{3}$$

where S_{ijk} is the normalization factor of kth replicate of jth time point in i^{th} protocol; λ_g is the normalized expression mean baseline for $i = j = 1$ (RPF sample in the first condition); u_{gi} is the fold change between two protocols with $u_{g1} = 1$; t_{gj} is fold change between two time points with $t_{g1} = 1$; w_{gij} is the fold change between the translation efficiency of two time points, with $w_{g11} = w_{g12} = w_{g21} = 1$; b_{gjk} denotes the one-to-one correspondence between RPF and mRNA samples, which is considered to be random. Note that the expression (3) is equivalent to a log-linear model if we express both sides in log scale, and the term w_{gij} corresponds to the interaction between protocol and time, which is of main interest.

Differential translation can be characterized by the dissimilarity between the changes in mRNA and RPF expressions across the two conditions. This is equivalent to determining whether the parameter w_{g22} in our model (3) differs significantly from 1. And the corresponding hypothesis test is

$$H^g_{w0} : w_{g22} = 1 \quad \text{vs.} \quad H^g_{w1} : w_{g22} \neq 1 \tag{4}$$

for each gene g.

We assume a hierarchical model for the gene-specific parameters $(\beta_g, \lambda_g, u_{g2}, t_{g2}, w_{g22}, b_{gjk})$ mentioned in equations (2) and (3). Since the simple null hypothesis we are most interested in is w_{g22}'s $H^g_{w0} : w_{g22} = 1$, and often times biologists are also interested in the differential expression analysis for RPF data between the two time points, which corresponds to testing whether the parameter t_{g2} differs significantly from 1, so a mixture of a point-mass at 1 and a Gamma distribution (due to conjugacy) is used as the hierarchical model for w_{g22} and t_{g2}. Again, we use Gamma distribution as the prior distribution for λ_g, u_{g2} and b_{gjk} because of their conjugacy,

$$
\begin{aligned}
& & \beta_g|\eta_\beta, \sigma^2_\beta &\stackrel{iid}{\sim} \mathrm{N}(\eta_\beta, \sigma^2_\beta) \\
&\text{Baseline:} & \lambda_g|\alpha_\lambda, \gamma_\lambda &\stackrel{iid}{\sim} \mathrm{Gamma}(\alpha_\lambda, \gamma_\lambda) \\
&\text{Protocol:} & u_{g2}|\alpha_u, \gamma_u &\stackrel{iid}{\sim} \mathrm{Gamma}(\alpha_u, \gamma_u) \\
&\text{Treatment:} & t_{g2}|\pi_{t1}, \alpha_t, \gamma_t &\stackrel{iid}{\sim} \pi_{t1}\delta_{\{1\}} + (1-\pi_{t1})\mathrm{Gamma}(\alpha_t, \gamma_t) \\
&\text{Translation Efficiency:} & w_{g22}|\pi_{w1}, \alpha_w, \gamma_w &\stackrel{iid}{\sim} \pi_{w1}\delta_{\{1\}} + (1-\pi_{w1})\mathrm{Gamma}(\alpha_w, \gamma_w) \\
&\text{Pairing:} & b_{gjk}|\alpha_b &\stackrel{iid}{\sim} \mathrm{Gamma}(\alpha_b, \alpha_b). \qquad (5)
\end{aligned}
$$

For computational convnience, we assume all the gene-specific parameters in (5) to be independent.

To formulate our problem of interest more clearly, we introduce latent variables Z_{gt} and Z_{gw} to denote which mixture component the time effect t_{g2} or translational efficiency change w_{g22} come from,

$$
\begin{aligned}
Z_{gt} &\sim \mathrm{Bernoulli}(1-\pi_{t1}), \\
Z_{gw} &\sim \mathrm{Bernoulli}(1-\pi_{w1}), \qquad (6)
\end{aligned}
$$

where π_{t1} and π_{w1} denote the chance that time effect and translation efficiency effects equal to 1. Thus, for each gene g, our main focus becomes

$$
H^g_{w0} : Z_{gw} = 0 \quad \text{vs.} \quad H^g_{w1} : Z_{gw} = 1. \qquad (7)
$$

Markov Chain Monte Carlo Simulation

There are $(11 + 2G)$ unknown parameters of interest in our model,

$$
\boldsymbol{\Theta} = \{\eta_\beta, \sigma^2_\beta, \alpha_\lambda, \gamma_\lambda, \alpha_u, \gamma_u, \alpha_t, \gamma_t, \alpha_w, \gamma_w, \alpha_b, \boldsymbol{t}_{g2}, \boldsymbol{w}_{g22}\}.
$$

The data likelihood function with observed data $\boldsymbol{Y} = \{Y_{1111}, Y_{1112}, \ldots, Y_{G222}\}$ is

$$
\begin{aligned}
L(\boldsymbol{\Theta}|\boldsymbol{Y}) =& \Pi^G_{g=1}\Pi^2_{j=1}\Pi^2_{k=1}\left\{[1-p_{g1jk}]^{(1-Z_{g1jk})} \cdot [p_{g1jk} \cdot f_{\mathrm{Poi}}(y_{g1jk}; \lambda_{g1jk})]^{Z_{g1jk}}\right\} \\
& \times \Pi^G_{g=1}\Pi^2_{j=1}\Pi^2_{k=1}\{f_{\mathrm{Poi}}(y_{g2jk}; \lambda_{g2jk})\} \\
& \times \Pi^G_{g=1}\{f_{\mathrm{N}}(\beta_g; \eta_\beta, \sigma^2_\beta)\} \\
& \times \Pi^G_{g=1}\{f_{\mathrm{Gamma}}(\lambda_g; \alpha_\lambda, \gamma_\lambda)\} \\
& \times \Pi^G_{g=1}\{f_{\mathrm{Gamma}}(u_{g2}; \alpha_u, \gamma_u)\} \\
& \times \Pi^G_{g=1}\left\{[\pi_{t1}]^{(1-Z_{gt})} \cdot [(1-\pi_{t1})f_{\mathrm{Gamma}}(t_{g2}; \alpha_t, \gamma_t)]^{Z_{gt}}\right\} \\
& \times \Pi^G_{g=1}\left\{[\pi_{w1}]^{(1-Z_{gw})} \cdot [(1-\pi_{w1})f_{\mathrm{Gamma}}(w_{g22}; \alpha_w, \gamma_w)]^{Z_{gw}}\right\} \\
& \times \Pi^G_{g=1}\Pi^2_{j=1}\Pi^n_{k=1}\{f_{\mathrm{Gamma}}(b_{gjk}; \alpha_b, \alpha_b)\}. \qquad (8)
\end{aligned}
$$

Here we propose a fully Bayesian analysis for the parameter estimation. For all unknown parameters in (5), due to either conjugacy or to reduce the complexity of computation of the posterior distribution, we assume independent non-informative priors as follows,

$$\begin{aligned}
\eta_\beta &\sim \mathrm{N}(0, 10^4) \\
\sigma^2_\beta &\sim \mathrm{IG}(0.001, 0.001) \\
\alpha_\lambda, \alpha_u, \alpha_t, \alpha_w, \alpha_b &\overset{iid}{\sim} \mathrm{Exp}(0.01) \\
\gamma_\lambda, \gamma_u, \gamma_t, \gamma_w &\overset{iid}{\sim} \mathrm{Gamma}(0.1, 0.1) \\
\pi_{t1}, \pi_{w1} &\overset{iid}{\sim} \mathrm{Unif}(0, 1).
\end{aligned} \tag{9}$$

After specifying the prior distributions, data will be used to update these prior distributions to obtain the posterior distributions for inference. Posterior inference in our proposed model is implemented by using Markov Chain Monte Carlo (MCMC) simulation (Tierney, 1994). Gibbs sampling (Geman and Geman, 1984) is the most commonly used tool to perform MCMC simulations for Bayesian hierarchical models. We applied MCMC Gibbs sampling method with Rjags to implement posterior inference.

From Bayesian inference, our null hypothesis or any quantities of interest could be easily obtained from the posterior distribution. For example, our null hypothesis of differential translation could be assessed through the posterior probability $p_g = Pr(Z_{gw} = 0|\boldsymbol{Y})$ for each gene g. In addition to the simple hypothesis testing problem, we could also applied our method to do other kinds of hypothesis testing, such as to test whether the fold change is within a certain interval or not.

Bayesian FDR Control

In ribo-seq studies, a large number of hypotheses are simultaneously tested, each relating to a gene. Hence, multiple testing procedures that control the number of false significant results is essential. False discovery rate (FDR) (Benjamini and Hochberg, 1995), defined as the expected proportion of false positives among the rejected hypotheses, has been the choice of error criterion in genomic studies.

The Bayesian version of FDR is an alternative way to estimate the FDR within the Bayesian framework. It has been proposed and discussed by several authors including Genovese and Wasserman (2003) and Newton *et al.* (2004). The Bayesian FDR can be obtained by using posterior probabilities of the null hypothesis. More specifically, for the analysis of translational efficiency change, for each gene g, $g = 1, \ldots, G$, we denote the posterior probability that gth null hypothesis is true by $v_g = P(w_{g22} \in \Delta_0|\boldsymbol{Y}_g)$. If we are interested in detecting differential translated genes, with $\Delta_0 = \{1\}$, $P(w_{g22} \in \Delta_0|\boldsymbol{Y}_g)$ is the posterior probability that gth gene is not differentially translated. $P(w_{g22} \in \Delta_0|\boldsymbol{Y}_g)$ can

be estimated by the proportion of the posterior samples obtained from MCMC for gene g that falls into the null set Δ_0, i.e.,

$$\hat{v}_g = \hat{P}(w_{g22} \in \Delta_0 | \boldsymbol{Y}_g) = \frac{1}{N} \sum_{m=1}^{N} I(w_{g22}^m \in \Delta_0 | \boldsymbol{Y}_g),$$

where N is the number of posterior samples. Then the posterior probability which the gth gene is differentially translated is estimated through $1 - \hat{v}_g$. We reject H_{w0}^g if the estimated posterior probability $\hat{v}_g$ is smaller than a critical value c^*. The critical value c^* is chosen based on controlling the FDR below a target level γ, for example, 0.05, i.e.,

$$c^* = sup\{c : \widehat{FDR}(c) < \gamma\},$$

where

$$\widehat{FDR}(c) = \frac{\sum_{g=1}^{G} \hat{v}_g I(\hat{v}_g < c)}{\sum_{g=1}^{G} I(\hat{v}_g < c)}.$$

So the Bayesian FDR controlled at level γ can be calculated by

$$\widehat{BFDR}(\gamma) = \frac{\sum_{g=1}^{G} \hat{v}_g I(\hat{v}_g < c^*)}{\sum_{g=1}^{G} I(\hat{v}_g < c^*)}.$$

Literature Cited

Benjamini, Y., Hochberg, Y.(1995). Controlling the false discovery rate: a practical and powerful approach to multiple testing. *J. R. Stat. Soc. B*, *57*, 289–300.

Geman, S., Geman D. (1984). Stochastic Relaxation, Gibbs distributions, and the Bayesian Restoration of ../figure. *Pattern Analysis and Machine Intelligence, IEEE Transactions*, *6*, 721–741.

Genovese, C., Wasserman, L. (2003). Bayesian and Frequentist Multiple Testing. *Bayesian Statistics*, *7*, 145–161.

Newton, M. A., Noueiry, A., Sarkar, D., Ahlquist, P. (2004). Detecting Differential Gene Expression with a Semiparametric Hierarchical Mixture Method. *Biostatistics*, *5*, 155–176.

Tierney L. (1994). Markov Chains for Exploring Posterior Distributions. *The Annals of Statistics*, *22*(4), 1701–1728.
