# Supplementary material for: Control of translation during the unfolded protein response in maize seedlings: Life without PERKs
Source: Plant Direct. 2020 Jul 30;4(7):e00241. doi: 10.1002/pld3.241 (PMC7390965; doi:10.1002/pld3.241)
Supplement: Supplementary file 3 — Supplementary Material [file PLD3-4-e00241-s003.pdf]

Editor's comments

RE: Control of Translation during the Unfolded Protein Response in Maize seedlings: Life without PERKs.

Dear Dr. Stephen Howell: dear Steve,

We have received reviews of your manuscript, which you may view at the end of this letter. On the basis of the advice received and my own evaluation, I sincerely regret that I must decline your manuscript for publication. The manuscript was re-evaluated by the same reviewers. Both reviewers were satisfied with a number of the changes, but Reviewer #1 restated significant concerns about both analysis and interpretation of the data. The comments received were discussed by both reviewers and I during a lengthy post-review consultation.

In looking over this revision, of what represents a long term project using challenging methodology and analyses, I found that I agree with the concern about a major conclusion, More specifically, it is not clear that the change in translational efficiency (TE) of the TU-upregulated mRNAs is highly variable. I am struggled with the lack of clarity of the normalization. I appreciate the efforts to sort out the relationship with change in TE and association wtih stress granules, but here there were also evident challenges. It is particularly difficult for me to render a decline for a second time given my own experience with these methods and interest in the relationship between stress responses, translational activity and mRNA preservation. I assure you that the two reviewers are equally disappointed that the outcome was not more favorable.

Given the policy of Plant Physiology to only allow one major revision, I find myself in the difficult position of letting you know that the journal will not consider any resubmission of this paper without a substantial additional research effort. I am sorry that I can't be more encouraging. (I am especially sorry given the current challenges faced by the research community due to the global health crisis).

Plant Physiology papers that are declined are often encouraged to transfer the paper and reviews to Plant Direct. That invitation is below. However, you may not wish to do so. You may either wish to make considerable changes and resubmit to Plant Physiology. Or, you may wish to send this to another journal. At this point I am very familiar with this submission and would be willing to handle it again as an editor or if asked to be a reviewer. I know you will be disappoited, but I agree with Reviewer 2 that further improvement guided by the comments

are needed before publication.

However, you may wish to consider publication in Plant Direct. Plant Direct has different publication criteria from those of Plant Physiology. You can transfer your manuscript and associated submission data automatically to Plant Direct via the link below.

Please note that transfer to Plant Direct does not guarantee acceptance of your manuscript, as the editors of Plant Direct will still evaluate the manuscript. Should you choose to transfer your manuscript, you will receive an email request from Plant Direct asking you to log in to the submission site's Author Center, provide any missing information unique to Plant Direct's submission process, and complete the submission.
If you wish to transfer your paper to Plant Direct, then click the link below:

https://pphys.msubmit.net/cgi-bin/main.plex?el=A4It6HXO4A5Tf1X5A9ftdC9hdCx2emZGpswsrOsJkQZ

Thank you again for the opportunity to consider your manuscript and review your work.

Sincerely yours,
Julia Bailey-Serres
Associate Editor, Plant Physiology

Responses to reviewers are indicated with a carot (>)

Reviewer #1 (Comments for the Author):

This revised manuscript describes how maize seedling roots respond to ER stress, as induced with the drug tunicamycin, at the level of translation. The work examines this response at multiple levels, formation of stress granules, polysome loading of mRNAs, ribosome footprinting, and association of mRNAs with stress granules. The work builds on a long and successful line of prior work by the same group, which characterized the unfolded protein response (UPR), including at the transcript level.

This is an interesting contribution because the UPR is a molecularly well defined cell biological concept that is elicited under various actual environmental stress conditions, and the authors have shown in prior work that they have a good

handle on studying its molecular cell biology. I also greatly respect that this an ambitious body of work.

The problem, as the manuscript lays out quite clearly, is that the various assays and responses yielded only partially congruent results. There are also continued concerns about the statistical treatment of the data, as the statistical model used is quite opaque, in my opinion. The effect sizes at the translation level are often small, which is not properly accounted for when inferences were made from the data. The emphasis on UPR genes is not well justified. Thus, this study presents a set of interesting observations (stress granule formation under tunicamycin, while bulk translation remains active, for example), accompanied by a series of also interesting trends (translational efficiency of UPR mRNAs, for example).

>Thanks for the various comments. We will address the congruent results in responses below. The statistical model was not described in detail in the revised submission because the paper has not yet been published. Therefore, we have described the statistical model in the revised text and have included the detailed model as a supplement. The emphasis on the UPR is justified. This paper is about the UPR, and we highlighted the UPR genes in various sections of the paper. We had published a previous paper in the Plant Cell showing that there is a surge in expression of the UPR genes in response to ER stress treatment and, therefore, we are concerned with the fate of those transcripts. It appears that the reviewer has interpreted this to mean that because of our interest in these genes that we assume that that they are being treated selectively. They are not. We had not been careful about pointing that out in our submission. But have made revisions in the text such that there should be no doubt about our position.

These observations do move the field forward and should be known to the community. However, they defy simple interpretation and generalization. Specifically, I take issue with several sweeping statements in the abstract that are too simplistic given the 'complexity' of the data, in my opinion.

>We assume that the most sweeping statement in the abstract to which the reviewer is referring is the statement "....... found a decline in translation efficiency, particularly among UPR genes, in response to ER stress." We have revised that to say " ..... found a decline in translation efficiency, including UPR genes, in response to ER stress." In the original statement "particularly among" we were stating our interest in these genes, not that they were selectively subject to a decline in efficiency. We have revised another statement in abstract to say: "The decline in translation efficiency is due to the fact that many mRNAs are not loaded onto polyribosomes (polysomes) in proportion to their increase in total RNA, instead some transcripts accumulate in stress granules (SGs)."

The authors have rewritten the paper and responded to comments in detail. I now understand better the choice of normalizing mRNA levels against actin and how it affects the data analysis. I appreciate that the abstract was revised to tone down certain claims. However, authors did not provide much in terms of the reanalysis and reinterpretation of the data that both reviewers had requested or encouraged. Fig. 3C and D are new, but only go so far to reconcile previous concerns. Thus I feel that several of the statements in the abstract are still not well supported by the data presented. I say this also in light of the responses to reviewer 2 on the polysome and stress granule analysis and confusion regarding the topic of 'UPR specificity'.

>It is the reviewers who have brought up UPR specificity and not the authors. Fig 3C and D are revised to show how the UPR genes show up in the context of all of the other genes. Note in Fig. 3D that many of the UPR genes fall to the right of the bulk of the other genes – not because of the way in which they are handled, but because these are genes that are upregulated in response to ER stress.

Before arguing my point in detail, let me propose that the data as presented in the figures may support an inference such as this: 'Bulk translational activity did not decline over 6 to 12 hours of ER stress. We performed multiple assays to measure translational efficiency of specific mRNAs including ribosome footprinting, polysome profiling, and physical association of mRNAs with stress granules. A number of UPR-induced mRNAs showed evidence of being partially sorted towards less translated cohorts in one or more of these assays. Because it is apparent that many other mRNAs follow similar trends, we conclude that the UPR is accompanied by a general reassortment of mRNAs between translated and nontranslated cellular pools.'

>Thank you for your contribution. Well stated!

Specific comments:

1. Abstract line 10: "We... found a decline in translation efficiency, particularly among UPR genes, in response to ER stress." Admittedly, Fig. 3B does show a global decline in translation efficiency.
a. And UPR genes indeed participate in this decline (Fig. 3D), but this being a global decline, there are many other mRNAs that are likewise affected. Thus, emphasizing UPR genes does not seem to be warranted. The authors replied to my earlier comment that they do not intended to single out UPR genes as specifically affected, but the abstract still emphasizes UPR genes.

>We will continue to emphasize the UPR genes, because as stated above, this is paper about the UPR. Again, just because we highlight the UPR genes does not mean that they are handled selectively. We are following them in the crowd of other mRNAs.

b. In Figure 3C, mRNAs such as ERDJ3a, BIP1 and bZIP60 are presented with nearly the same response at the RNA level as at the RPF level. Thus at least these (and other) UPR mRNAs have either no translational decline or a decline close to the median of all mRNAs.

>Not so. In Fig 3C, note that ERDj3a, Bip1 and Bip2 are well above the regression line, not on the regression line like most of the other RNAs.

c. There are discrepancies how statistical significance is reported. In line 184 " The number of genes with significant change in translation efficiency (FDR<0.05) was plotted against log2 fold change in RNA abundance (Figure 3A) and log2 fold change in translation efficiency (Figure 3B)." This is difficult to comprehend, but what the figure shows is simply the distribution of log2 fold changes in RNA abundance (3A) and translation efficiency (Fig. 3B). A casual reading of this passage suggests that all data shown passed FDR, but this is difficult to believe and probably a clerical error. Clearly, if there was no change in RNA abundance, how can this change be significant and pass an FDR threshold?

>The significance is in reproducibility between reps.

d. The description of the statistical model used to define changes in translational efficiency (TE) is not very transparent. Has this method been validated for translation data or footprint data (the Newton et al. reference given is highly cited but I could not find examples for this). I appreciate the detailed response to my prior comment. I worry that other readers who do not have access to the response (and open Dataset 3) will be bewildered, also considering that many readers know as little about statistical modeling as I do. From the authors' response I gathered that the modeling technique involves defining certain genes as having no change in TE, and these genes are then declared as "confidently unchanged" and therefore pass the FDR threshold even though they have no change in TE (?). Some clarification to dispel my skepticism about this should be added in the Methods. This may also entice more people to give this miraculous method a closer look. In any event, please help the readers understand the meaning of FDR under this model's assumptions as compared to an FDR in parametric statistics. I think it is not asking too much to provide us with results

from an alternate, robust, parametric analysis such as ANOVA and a volcano plot, as previously requested.

We have included a new paragraph in the methods about the statistical treatment and provide in the supplemental material a full description of the statistical method.

e. If all the TE data in Fig. 3D (gray datapoints) are statistically validated, then why not analyze them, especially in light of prior reviewers' requests? As presented the analysis is incomplete.

>We are not interested in analyzing all of the genes. We have highlighted some of the UPR genes, because again, this is paper about the UPR.

2. Abstract line 11: " The decline in translation efficiency is due to the fact that many of the new mRNAs are not loaded onto polyribosomes (polysomes)." According to the data (Fig. 3D trendline), a 4-fold increase in RNA level is accompanied by a two-fold decline in translation efficiency; and for ERDJ3a, an 8-fold increase in mRNA is accompanied by a small (1.5-fold) decline in TE (line 208). I would conclude from this that the majority of newly synthesized mRNA molecules enter into polysomes. The abstract needs to be revised to reflect the data more accurately.

>The data in Fig. 5A simply says that some of the UPR genes such as BIP2 and ERdj3a are not loaded onto polysomes in proportion to their increase in total RNA. That is consistent and congruent with the data in Figure 3C.

3. Abstract line 12: "... instead (the UPR-induced mRNAs) are diverted to accumulate in stress granules (SGs)." The data (Fig. 5A and 5C) show that for two of the heavily induced UPR mRNAs, BIP2 and ERDJ3a, the polysome loading drops dramatically and a portion ends up in stress granules. This supports the main message, but what about other UPR mRNAs, especially those with low RPF counts such as Derlin1 and PDI-1 whose polysome loading did not change? The abstract should reflect this variation between different assays. It paints an overly clear picture when in fact the data are not. The inference that UPR induced transcripts end up in stress granules and undertranslated is supported by mixed evidence. Different UPR genes have evidence from different assays (Fig. 3C, Fig. 5). Not all data are congruent.

>Yes, BIP2 and ERDj3a, two of the strongest UPR markers behave as we describe. There may be other reasons why the increase of Derlin1 in polysomes is closer to its increase in total RNA (Fig. 5A). For example, it is possible that the polysomes bearing Derlin1

transcripts are less loaded with ribosomes in response to ER stress.

4. Abstract line 13: "The RNAs that populate SGs are not derived from the disassembly of polysomes because protein synthesis remains steady during stress." I agree that the puromycylation assay and polysome profiles show that translation remains very active during stress. However, the data are equally consistent with the alternative hypothesis, namely that some pre-existing mRNAs drop out of polysomes and are replaced by newly synthesized mRNAs induced during the UPR. The abstract needs to reflect this uncertainty. With more time, this could be discussed in much more detail. One could address this question experimentally by checking what happens to generally unstable mRNAs during the UPR? Do these mRNA, which are constantly being transcribed, also end up preferentially in stress granules and at low TE? The data in the bottom portion of Fig. 3D or additional SG-Q-PCR etc. could address this.

>We tried to measure mRNA stability for different reasons. We wanted to how the levels of UPR mRNAs were maintained as the surge in accumulation subsides. However, we found that actinomycin D and cordycepin are not taken up by the seedlings.

Other comments on main text:

5. Fig. 4B is supposed to be an immunoblot but legend does not speak of antibodies or any similar binding assay and instead describes a covalent derivation reaction (click chemistry?).

>Yes, it does. The legend says "immunoblotted with an antibody to puromycin."

6. The discussion dwells a lot on mammalian SG formation and how SG may form in plants. The paper itself does not really inform this discussion, which seems to be a tangent. For example, the paper does not give any numerical estimate of the 'surge' (bulk increase) in mRNAs during the UPR. +10%, or +100%? Since this is so important for the line of argumentation in the paper, and since they have the data in hand, why not tell us? - It would be useful to discuss the discrepancies between the different assays, why some mRNAs decline in footprints while remaining in polysomes (their ribosome loading may simply decline). And why are mRNAs found in the SG fraction when there are apparently no SGs (at time 0). It would be useful to discuss that SG data come from protoplasts, which is something I missed until reading the response to review.

>The data set for the upregulation of the UPR genes is in the Plant Cell paper by Srivastava et al. (2018). There are substantial differences in the fold increase in expression of the different genes. We show many of the genes as examples in

graphs in the paper, but one can look at the data sets submitted with the paper. For the RNA population as aa whole, see Figure 3A. As for SGs, there is some background RNA content in the SG-enriched fraction at zero time. These are SG enriched fractions and not affinity purified SGs. The legend says "B. Confocal images of maize leaf protoplasts."

7. Please mention in the paper what was the background set in the GO analysis. Please note that your GO analysis will identify all functional categories that are enriched in roots versus whole genome background set. This is why using 'genes expressed in my experiment' as the background set is more informative.

> We now indicate "The background used by AgriGO was locus ID v3.30 (Gramene release 50). "

8. Raw data have still not been provided. Dataset 2 is in acceptable form but Datasets 1 and 3 only provide averages and are incompletely annotated. Please add a sheet with Legend for column labels etc.

>Data sets have been provided and will be submitted to GEO if the paper is accepted for publication.

Reviewer #2 (Comments for the Author):

I appreciate the effort of the authors submitting this new manuscript. I like this new one much better. Although I would still like to see the experiments I suggested, I understand the challenges but wish they would overcome them.

Regarding the origin of the monosomal peak in the profiles, of course I know they come from unengaged polysomes... it was more a question out of curiosity. I intended to ask why are there more subunits in the TM treatment than in the control experiment. But if those peaks are not reproducible, it is possible that they don´t have a biological meaning.

>The balance between subunits and monosomes seemed somewhat variable from run to run, so we were not sure if it had biological meaning or whether this was simply a technical matter.

I am, in general, satisfied the author´s answers to my questions. However, I am still concerned about the fact that the fact of the ribo-seq and rna-seq reads, in different replicates and different time points pile up exactly at the same parts of the gene. Despite the author´s answer, I am quite familiar with ribo-seq reads, and never seen them piling up at exactly the same places of the ORF. I think there

might be some issue with the libraries that might alter the interpretation of the ribo.seq analysis data. However, as both the profiles and the Sunset experiment don´t show any dramatic change in translation, I believe the result, but still think that something is going on that should be revised in future experiments.

>We have removed that figure. While it does show that ribosomes are distributed along the mRNA and piling up at various places, if there was a block in translation initiation, we would not expect to see a piling up at the transcription start site.

Typos:
L132: in response to stresses, such AS heat, ...
L211: scAtterplot

>Corrected both
